# Self-Chemiluminescence-Triggered Ir(III) Complex Photosensitizer for Photodynamic Therapy against Hypoxic Tumor

**DOI:** 10.1021/acs.inorgchem.4c02399

**Published:** 2024-08-16

**Authors:** Shengnan Liu, Haoran Chen, Qi Wu, Yan Sun, Yu Pei, Ziwei Wang, Dongxia Zhu, Gungzhe Li, Martin R. Bryce, Yulei Chang

**Affiliations:** † Key Laboratory of Nanobiosensing and Nanobioanalysis at Universities of Jilin Province, Department of Chemistry, Northeast Normal University, 5268 Renmin Street, Changchun, Jilin Province 130024, P. R. China; ‡ State Key Laboratory of Luminescence and Applications, Changchun Institute of Optics, Fine Mechanics and Physics, Chinese Academy of Sciences, Changchun, Jilin Province 130033, P. R. China; § Jilin Provincial Science and Technology Innovation Center of Health Food of Chinese Medicine, Changchun University of Chinese Medicine, Changchun, Jilin Province 130117, P. R. China; ∥ Department of Chemistry, Durham University, Durham DH1 3LE, U.K.

## Abstract

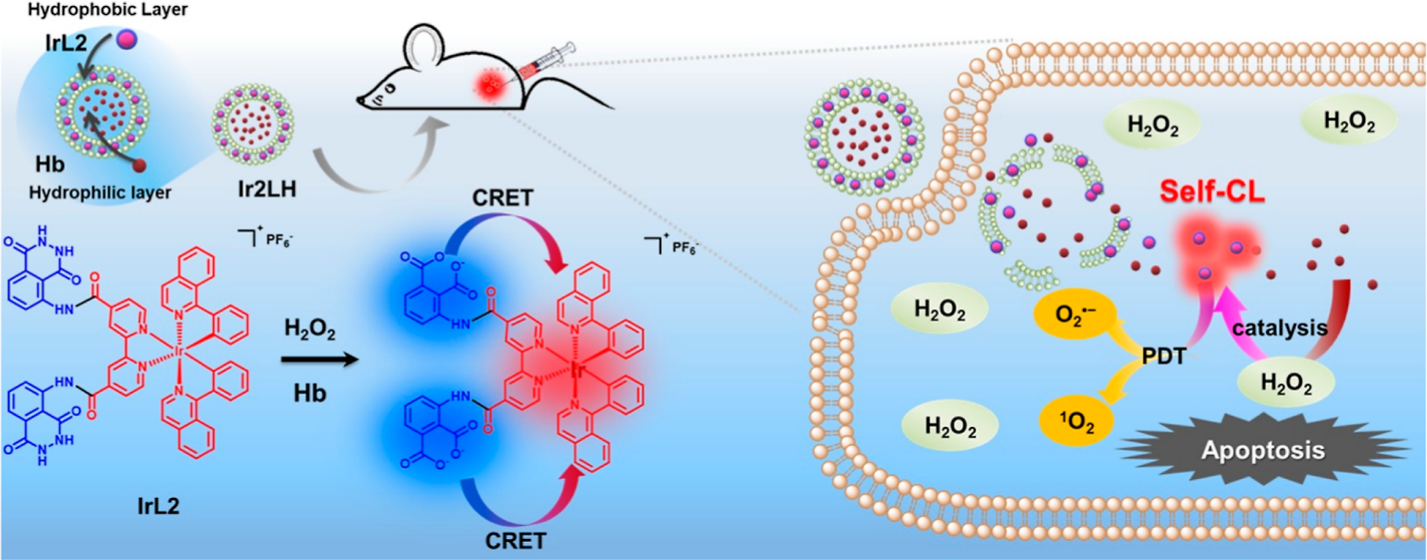

The limited optical penetration depth and hypoxic tumor microenvironment (TME) are key factors that hinder the practical applications of conventional photodynamic therapy (PDT). To fundamentally address these issues, self-luminescent photosensitizers (PSs) can achieve efficient PDT. Herein, a self-chemiluminescence (CL)-triggered Ir complex PS, namely, **IrL2**, with low-O_2_-dependence type I photochemical processes is reported for efficient PDT. The rational design achieves efficient chemiluminescence resonance energy transfer (CRET) from covalently bonded luminol units to the Ir complex in **IrL2** under the catalysis of H_2_O_2_ and hemoglobin (Hb) to generate O_2_^•–^ and ^1^O_2_. Liposome **IrL2H** nanoparticles (NPs) are constructed by loading **IrL2** and Hb. The intracellular H_2_O_2_ and loaded Hb catalyze the luminol part of **IrL2H**, and the **Ir2** part is then excited to produce types I and II reactive oxygen species (ROS) through CRET, inducing cell death, even under hypoxic conditions, and promoting cell apoptosis. **IrL2H** is used for tumor imaging and inhibits tumor growth in 4T1-bearing mouse models through intratumoral injection without external light sources. This work provides new designs for transition metal complex PSs that conquer the limitations of external light sources and the hypoxic TME in PDT.

## Abstract 2

The application of Ir complex photosensitizers with self-chemiluminescence properties for photodynamic therapy (PDT) is novel and, to our knowledge, has not been reported until now. This work provides a versatile precedent for using functionalized transition metal complexes to self-generate chemiluminescence for PDT, thereby extending the molecular platform for tackling the enduring problems of an external light source and the hypoxic tumor environment in clinical applications. In vitro and in vivo experiments are reported.

## Introduction

Photodynamic therapy (PDT) has attracted widespread interest as a clinical method for treating cancer due to its noninvasive nature, high spatial selectivity, and limited side-effects.^1−5^ In a typical PDT process, a specific wavelength of external light from a laser or a light emitting diode (LED) activates a photosensitizer (PS) from its singlet ground state (S_0_) to the first excited singlet state (S_1_), which is converted to the excited triplet state (T_1_) through intersystem crossing (ISC). The excited PS then interacts with oxygen-containing substrates to generate cytotoxic reactive oxygen species (ROS), which oxidize and damage biomolecules such as DNA, proteins, lipids, and carbohydrates, inducing tumor cell apoptosis.^5−7^ Unfortunately, owing to the significant absorption and scattering of the incident light by tissues, the practical applications of PDT suffer from limited light penetration depth [visible light ∼3 mm; near-infrared (NIR)-I light ∼1 cm; and NIR-II laser ∼3 cm] and potential tissue overheating during long-term imaging.^6,8−12^ Therefore, PDT is not usually a first-line therapeutic method, and there is an urgent need to develop new PSs that do not require external light excitation for PDT, especially in deep tissues.

Chemiluminescence (CL) is a process whereby a molecule is activated by chemical oxidation and then transfers its energy to an adjacent fluorophore, the excited state of which returns to its ground state, accompanied by luminescence. Chemiluminescence resonance energy transfer (CRET) can achieve in situ PDT with self-luminescent PSs that overcome the autofluorescence interference and penetration depth limitation of external light sources.^8,13−24^ Luminol, as a classical chemiluminescent reagent, is converted in a reaction sequence that is catalyzed by H_2_O_2_ and metal ions to yield excited-state aminophthalate, which emits blue light.^13,25^ One of the features of the tumor microenvironment (TME) is overexpression of H_2_O_2_, which is obtained from superoxide ions generated by mitochondria in a process that is catalyzed by the enzyme superoxide dismutase.^26^ Therefore, H_2_O_2_ can be an ideal trigger to serve as a catalyst for luminol CL, so that the CL can be generated in situ within the diseased tissue without the need for an external light source to drive the PDT.^26^ In addition, the ROS production of CL-triggered PDT is dependent on the CRET efficiency, which complies with the Förster resonance theory, that is, the efficiency of energy transfer is inversely proportional to the sixth power of the donor–acceptor distance.^27,28^ Therefore, covalent bonding between the chemiluminescent donor and PSs should enhance PDT compared to simply mixing the two components in a blend where they would be distributed randomly and PDT would rely on intermolecular energy transfer processes.^29−31^ However, there are only a few reports of covalently linking luminol with PSs, namely, chlorin (Ce6)^32−34^ and boron-dipyrromethene (BODIPY)^9,35^ for CRET-mediated PDT without needing an external light source. These PSs are confined to the traditional type II PDT process, which has high O_2_ dependence, and their use is restricted by the hypoxic TME, which arises due to rapid tumor growth and insufficient oxygen supply.^36,37^

Alternative strategies for PDT have been explored including O_2_ delivery and blood circulation enhancement to address the limitation of hypoxia; however, their efficacy is not ideal due to fast O_2_ efflux and complicated operation.^38,39^ The type I process has low O_2_ dependence, which is superior for PDT with the PSs reacting with intracellular substrates to generate cytotoxic ROS, such as O_2_^•–^ and ^•^OH.^40,41^ CL-triggered PSs with type I photochemical properties urgently need further research. Metal-complex PSs with versatile electronic excited state properties are known to achieve type I photochemical processes via their promoted electron transfer pathways and high rates of ISC.^42,43^ For examples, coumarin derivatives have been covalently linked to the ligands of cyclometalated Ru(II)^44^ and Ir(III) complexes.^45^ However, these conjugates required external light irradiation to effect type I PDT. Ir complex PSs with excellent photostability, outstanding photophysical properties, efficient ISC ability, and tunable ligand structures have attracted increasing attention.^46−50^ Most importantly for the present work, Ir complexes can absorb in the blue region,^43,51,52^ which overlaps well with the fluorescent donor luminol. We, therefore, postulated that Ir complexes could produce ROS through a luminol-mediated CRET process. Applying Ir complex PSs with self-CL properties for PDT is novel and, to our knowledge, has not been reported until now.

**Scheme 1:**
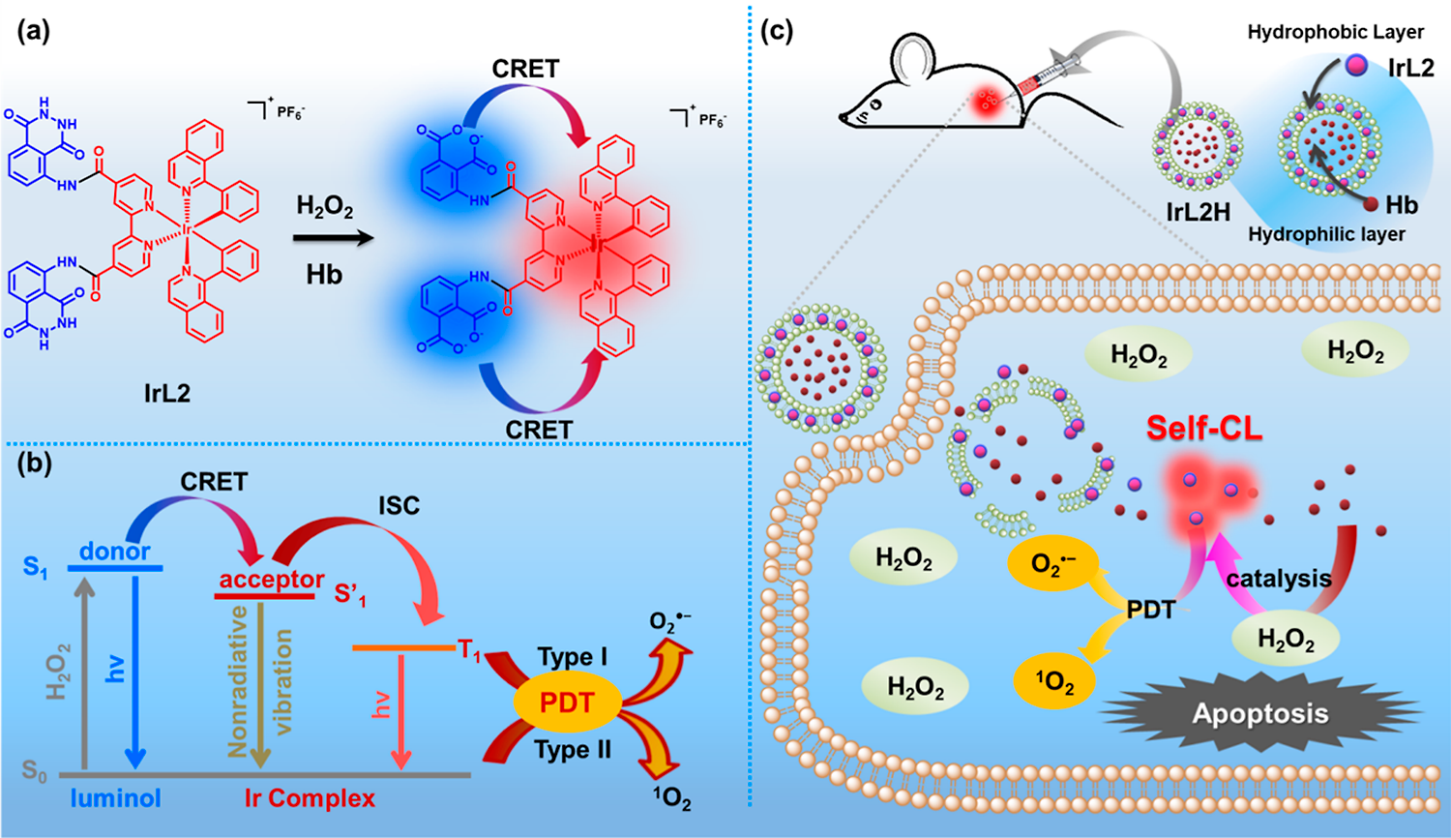
Schematic Diagram of the **IrL2** Structure and Self-CL Triggered PDT Using **IrL2H** NPs The bright blue and red colors in panel (a) represent the luminescence colors of the CL of luminol and the Ir center. (b) CL-triggered PDT mechanism of **IrL2**. (c) Schematic diagram of the CL-triggered in vitro and in vivo PDT process using **IrL2H** NPs

Herein, by rational choice of ligands, the blue-light-absorbing Ir complex, **Ir2**, with carboxyl groups on the N^N auxiliary ligand, was combined with luminol through amide condensation reactions to obtain self-CL triggered PSs, **IrL2** (Scheme 1). **IrL2** achieves CL under H_2_O_2_ and hemoglobin (Hb) catalysis, producing O_2_^•–^ and ^1^O_2_. The mechanism is that H_2_O_2_ and Hb catalyze the CL of the luminol units, exciting the Ir complex through CRET and stimulating the Ir complex to produce types I and II ROS through electron and energy transfer, respectively. Liposome-based **IrL2H** nanoparticles (NPs) were constructed based on **IrL2** and Hb for in vitro and in vivo experiments. It is shown that with the catalysis of intracellular H_2_O_2_ and loaded Hb, luminol units excite the **Ir2** core to produce type I and II ROS through CRET; **IrL2H** promotes cell apoptosis and even induces cell death under hypoxic conditions. In vivo, **IrL2H** achieves tumor imaging within 3 h and inhibits tumor growth in 4T1-bearing mouse models through intratumoral injection. This work opens a new way to address the problems inherent with an external light source and the hypoxic TME limitation in PDT.

## Results and Discussion

### Design and Synthesis of **Ir1**, **Ir2**, **IrL1**, and **IrL2**

**Scheme 2:**
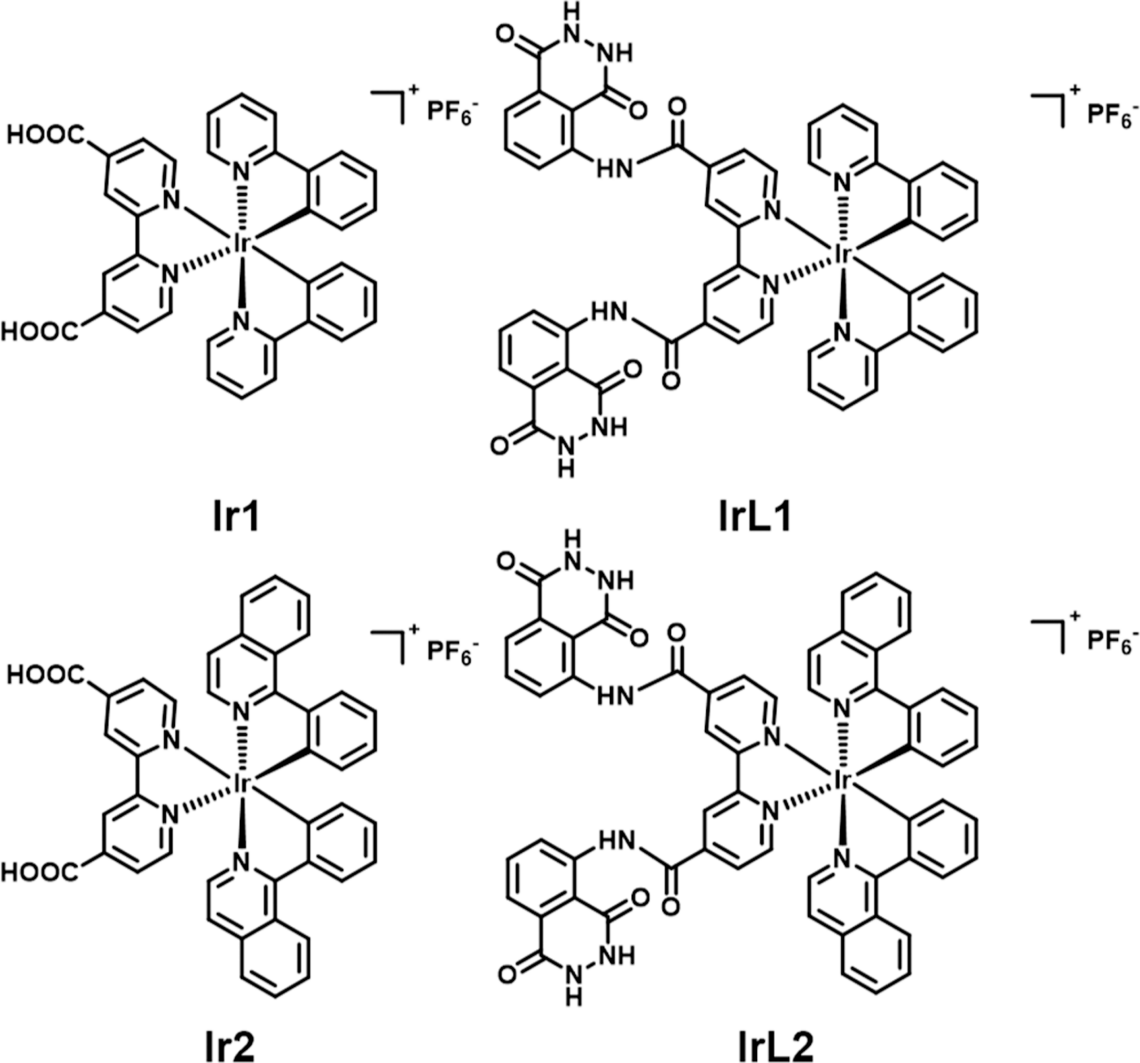
Structures of **Ir1**, **IrL1**, **Ir2**, and **IrL2**

Two Ir complexes **Ir1**(53) and **Ir2** were synthesized using different C^N ligands (2-phenylpyridine and 1-phenylisoquinoline for **Ir1** and **Ir2**, respectively) and an N^N ligand substituted with two carboxylic acid groups. The amino group of luminol reacted with the carboxyl groups of **Ir1** and **Ir2** through amide condensation to obtain **IrL1** and **IrL2**, respectively. The structures of **Ir1**, **IrL1**, **Ir2**, and **IrL2** are shown in Scheme 2, and their syntheses are shown in Scheme S1 in the Supporting Information. The molecular structures were confirmed by ^1^H NMR, ^13^C NMR, and ^19^F NMR spectroscopy, mass spectrometry, high-performance liquid chromatography (HPLC) (Figures S1–S11), and elemental analysis.

### Photophysical Properties and ROS Generation Ability of **Ir1** and **Ir2**

The photostability of **Ir1** and **Ir2** was tested to avoid the possible impact of photobleaching during the PDT process.^54,55^ The CL of luminol is mainly in the blue region, about 425 nm. Therefore, a 425 nm LED was used as the light source to monitor the photostability of **Ir1** and **Ir2** during 15 min of irradiation. The results showed no significant change in the absorption spectra of **Ir1** and **Ir2** (Figure S12), indicating their excellent photostability.

**Figure 1:**
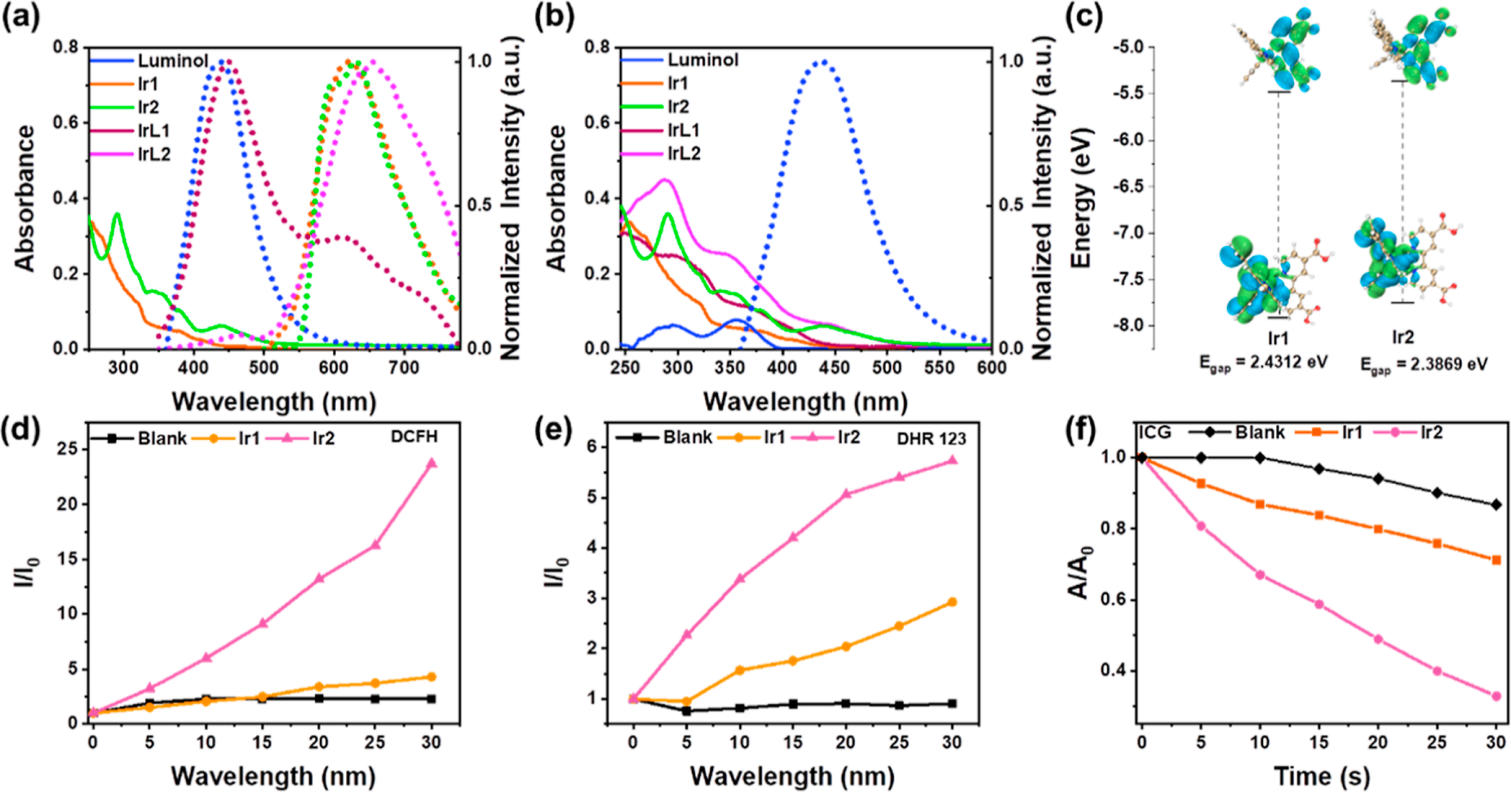
(a,b) UV–vis absorption spectra (solid lines) and normalized PL spectra of **Ir1** and **Ir2** and normalized CL spectra of luminol, **IrL1**, and **IrL2** (dashed lines) (1.0 × 10^–5^ M in DMF solution). (c) Energy level distribution and energy gap of the HOMO (lower structures) and LUMO (upper structures) in **Ir1** and **Ir2**. Change of PL intensity of DCFH (d) and DHR 123 (e) with the change of time under different conditions. *I*_0_ = initial intensity of 525 nm. *I* = real-time intensity of 525 nm with various times of light exposure. (f) Decay rates of ICG under different conditions. *A*_0_ = initial absorbance of 790 nm. *A* = real-time absorbance of 790 nm with various light exposure time. Light: 425 nm, 20 mW cm^–2^.

To determine the potential of the Ir complexes to connect with luminol to construct PSs, their photophysical properties and ROS generation abilities were explored. As shown in Figures 1a,b, and Table S1, the absorption of **Ir1** and **Ir2** is mainly in the ultraviolet and blue-violet regions, and they emit red light with λ_max_ of 615 and 632 nm, respectively, representing a large Stokes shift, which is beneficial for reducing the signal-to-noise ratio in practical therapeutic applications. The absorption of **Ir1** has a limited overlap with the emission of luminol, which suggests only limited energy transfer efficiency in the CRET process. On the contrary, **Ir2** shows a significant red shift in absorption, which overlaps well with the emission of luminol at 400–500 nm (Figure 1a,b), indicating its great potential for combining with luminol to construct an efficient CL material.

Density functional theory (DFT) calculations showed that the highest occupied molecular orbital (HOMO) of **Ir1** and **Ir2** is distributed in the cyclometalated C^N ligands and the metal Ir center. In contrast, the lowest unoccupied molecular orbital (LUMO) is distributed on the auxiliary N^N ligand (Figure 1c), indicating that these two complexes have excellent intramolecular charge transfer (ICT) characteristics, which can enhance the charge transfer state energy and further promote ROS production.^56^ Compared to **Ir1**, the π-extended C^N ligands of **Ir2** increase the electron conjugation within the complex, reduce the HOMO–LUMO gap, increase the electronic transition ability, and cause a red shift in the absorption spectra (Figure S15), which is consistent with the experimental results.

To further verify the ROS generation from **Ir1** and **Ir2** under 425 nm irradiation, 2,7-dichlorofluorescencein (DCFH) was used as an indicator. As shown in Figures 1d and S16, both **Ir1** and **Ir2** generate ROS within 30 s of irradiation, and **Ir2** has an excellent ability to produce ROS. Dihydrorhodamine 123 (DHR-123) was used as an indicator to detect the type I ROS produced by **Ir1** and **Ir2** (Figures 1e and S17). Both complexes produce type I ROS upon 425 nm irradiation, with **Ir2** showing a superior ability. Similar results were observed from the electron paramagnetic resonance (EPR) detection using 5,5-dimethyl-1-pyrroline-*N*-oxide (DMPO) as an indicator (Figure S19). After the irradiation of **Ir1** and **Ir2**, DMPO was added and the DMPO–OOH^•^ adduct was registered, giving a spectrum consisting of a quadruplet, which indicates the generation of O_2_^•–^ in the solution.^57,58^ Indocyanine green (ICG) was used to assess type II ROS production (Figures 1f and S20–S23). Compared with the control group, **Ir2** reduced the absorption of ICG at 790 nm to below 40% within 30 s under 425 nm irradiation. The quantum efficiency of singlet oxygen generation was calculated to be 0.69 for **Ir1** and 0.79 for **Ir2**, demonstrating the excellent capacity of **Ir2** to produce ^1^O_2_. Calculations analyzed the effect of the functional groups of the two Ir complexes on their excited states. At the level of TD-B3LYP/6-31G(d) SMD (solvent = H_2_O), the singlet–triplet energy difference of the two complexes is relatively small (Figure S24), and the degeneracy of the **Ir2** excited states significantly increases at lower energy levels, which enhances its ISC channel and further optimizes its ISC ability, promoting the production of triplet excitons and thus improving its ROS generation ability. Therefore, **Ir1** and **Ir2** are type I and II PSs, respectively; especially, **Ir2** has enhanced ROS production ability under 425 nm irradiation compared to **Ir1**, which provides experimental support for the application of **Ir2** in PDT.

### Chemiluminescence Behavior of **IrL1** and **IrL2**

Given the ROS production ability of **Ir1** and **Ir2** and the overlap in their absorption spectra with luminol emission, chemiluminescent molecules **IrL1** and **IrL2** were synthesized. Their photophysical properties are shown in Figure 1a and Table S1. The absorption spectra of **IrL1** and **IrL2** contain the expected features of both luminol and Ir complexes. Luminol is mainly converted into excited 3-aminophthalic acid in the presence of H_2_O_2_ catalyzed by reagents such as hemoglobin (containing Fe^2+^) or catalase enzyme.^59,60^ The excited state relaxes to the ground state with the emission of light. Therefore, H_2_O_2_ and Hb were used to activate the CL of **IrL1** and **IrL2**. As shown in the CL spectra in Figure 1a, the blue emission peak of **IrL1** is from luminol, while the red emission peak is from the luminescence of luminol transferred to **Ir1** through CRET. The emission of **IrL2** at 635 nm is mainly from the **Ir2** unit. This derives from the excellent overlap between the absorption of **Ir2** and the emission of luminol, resulting in a more efficient CRET process (compared to **IrL1**), and the emission in the blue region is negligible. The design strategy has successfully achieved efficient CRET for realizing self-CL.

**Figure 2:**
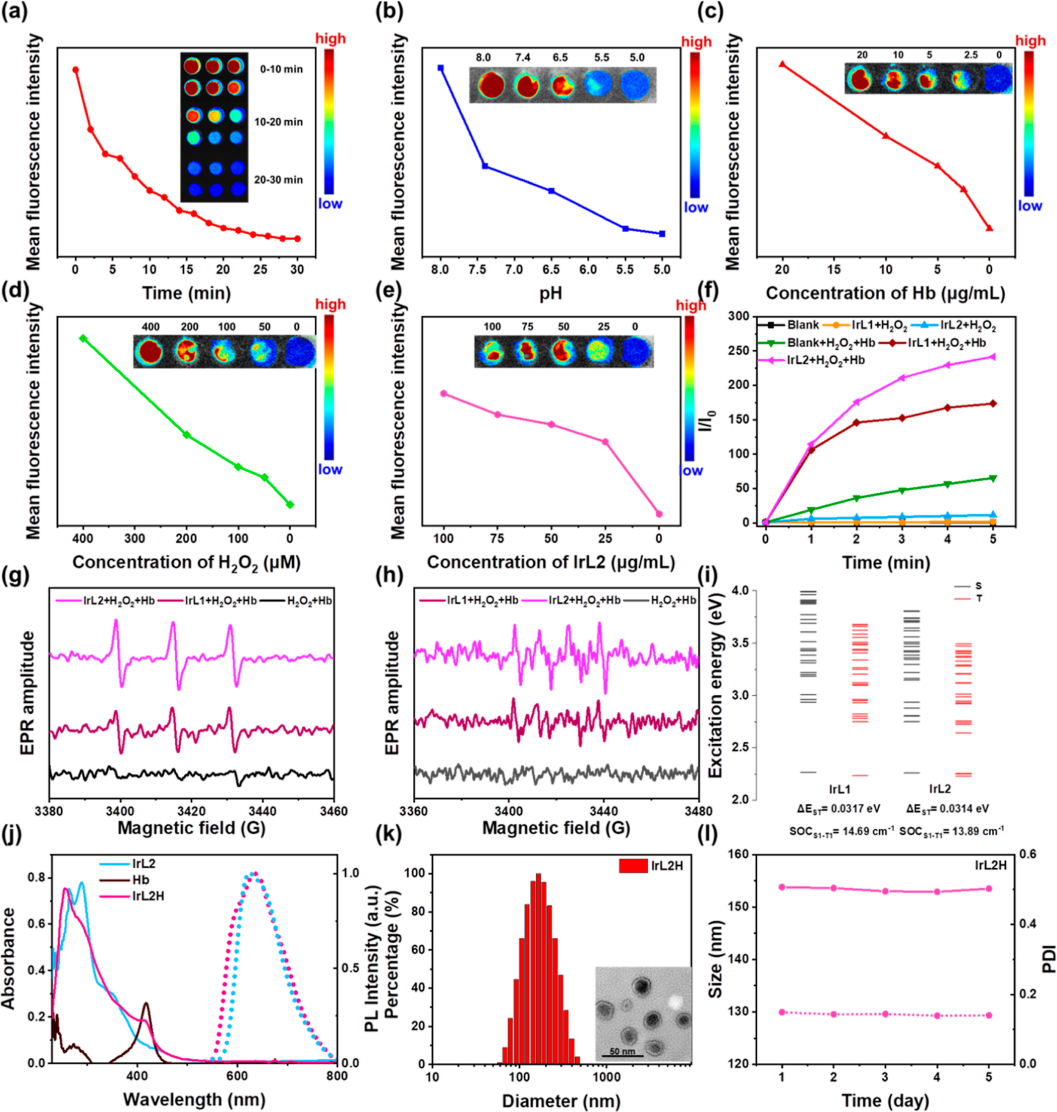
(a–e) Average fluorescence intensity of **IrL2** with different treatments. Inset image: CL image of **IrL2** with different treatments. (f) Change of PL intensity of DCFH with the change of time under different conditions. *I*_0_ = initial intensity of 525 nm. *I* = real-time intensity of 525 nm with various times. EPR signals of TEMP (for type-II ROS detection) (g) and DMPO (for type-I ROS detection) (h) with different treatments in MeOH solution. (i) Singlet and triplet energy levels of **IrL1** and **IrL2** and their spin–orbit coupling values with S_1_–T_1_ and S_0_–T_1_ transitions. (j) Solid line: absorption spectra of **IrL2**, Hb, and **IrL2H**; dashed line: PL spectra of **IrL2** and **IrL2H**, λ_ex_ = 425 nm. (k) DLS data for the NPs of **IrL2H**. Inset image: TEM image of **IrL2H**. (l) Changes in the size (solid line) and PDI (dashed line) of **IrL2H** in 5 days.

Subsequently, more detailed studies were conducted on the CL behaviors of **IrL1** and **IrL2**. The effects of different materials, reaction time, pH, Hb concentration, H_2_O_2_ concentration, and **IrL2** concentration were investigated in black 96-well plates. As shown in Figure S25, the CL of **IrL2** is significantly brighter than that of **IrL1** under the same catalytic conditions. **IrL2** CL is time-dependent, and its emission intensity decreases over time (Figure 2a). This result is similar to previous reports on luminol.^33,34^ The effect of pH on the CL of **IrL2** was investigated by adding the same concentration of **IrL2**, H_2_O_2_, and Hb to phosphate-buffered saline (PBS) solutions with different pH values (Figure 2b), indicating that the CL of **IrL2** almost disappeared at pH 5.0 and gradually enhanced with increasing pH. **IrL2** emitted CL at pH = 6.5 (about the typical pH in the TME),^61,62^ which favors its application in cancer cells. The effect of catalysts on **IrL2** CL was explored by changing the Hb concentration; with increasing Hb concentration, the CL gradually increased, which means that the Hb concentration has a significant impact on the CL of **IrL2** (Figure 2c). Hence, in the subsequent preparation of NPs, Hb was added as the catalyst in the system to convert luminol to excited-state aminophthalate in situ. In addition, Figure 2d indicates that the CL of **IrL2** gradually enhances with the increase in the H_2_O_2_ concentration. Usually, there is about 100 μM H_2_O_2_ in cancer cells,^26,63^ and according to our experimental results, this concentration is sufficient to activate the CL of **IrL2** for application in cell experiments. The effect of the **IrL2** concentration on its CL is not very significant within the range tested (Figure 2e). Therefore, cell experiments can be conducted within this concentration range. Moreover, the comparison of CL ability between covalently bonding the compounds and simply mixing the two components is shown in Figure S26. It is obvious that the covalently linked **IrL2** exhibits stronger luminescence than simple (noncovalent) mixtures of **Ir2** and luminol, indicating the higher CRET efficiency of the covalently bonded compounds. The above results indicate that the CL of **IrL2** gradually increases with the increase of pH, Hb content, and H_2_O_2_ concentration and could be achieved under appropriate TME pH and H_2_O_2_ concentrations. These data provided the basis for subsequent cell experiments.

### CL-Excited ROS Generation Ability of **IrL1** and **IrL2**

To further verify the possibility of CL Ir complexes for PDT, the ROS production by CL-excited **IrL1** and **IrL2** was investigated As shown in Figures 2f and S28, DCFH was used to detect the ROS in the solution under different conditions. Compared with that of the control group, the emission of DCFH gradually increased with time after adding H_2_O_2_ to the solutions of **IrL1** and **IrL2**. This may be because H_2_O_2_ catalyzes the conversion of the luminol part of the PSs to the excited state and then excites the Ir complexes through CRET to produce ROS. More ROS are generated after adding H_2_O_2_ to the solution of **IrL2**, compared to **IrL1**, which is due to its the superior CRET with **IrL2**. Therefore, Hb should also enhance ROS production by **IrL1** and **IrL2**. To verify this hypothesis, the ROS production of **IrL1** and **IrL2** in the presence of H_2_O_2_ and Hb was detected simultaneously. A blank solution with H_2_O_2_ and Hb was monitored, and the emission spectra were obtained of DCFH in **IrL1** and **IrL2** solutions in the presence of the same concentrations of H_2_O_2_ and Hb, respectively. Compared with the blank solution (green line in Figure 2f), the emission of DCFH in **IrL1** and **IrL2** solutions was significantly enhanced, and the curve flattened over time. The data show that especially **IrL2** has an excellent ability to produce ROS under catalysis by H_2_O_2_ and Hb, again revealing its superiority over **IrL1** for self-CL-triggered PSs.

2,2,6,6-Tetramethylpiperidine (TEMP) and DMPO were used as indicators to detect the type II and type I ROS production, respectively, by EPR spectroscopy (Figures 2g,h, and S29). Compared with those of the blank control group, the spin signals of the TEMPO^•^ adduct in **IrL1** and **IrL2** solutions were significantly enhanced to give a spectrum consisting of a triplet, with 1:1:1 relative intensities after the addition of H_2_O_2_ and Hb (Figure 2g) which indicate the generation of ^1^O_2_.^64,65^ Significant DMPO–OOH^•^ adduct signals of a quadruplet with 1:1:1:1 relative intensities were also observed for **IrL1** and **IrL2** after mixing with H_2_O_2_ and Hb (Figure 2h), consistent with the production of O_2_^•–^.^66,67^ The signals of TEMPO^•^ and DMPO–OOH^•^ for the **IrL2** solution were more obvious than for **IrL1**, as shown in Figure 2g,h, confirming the excellent CL-induced type I and II ROS production ability of **IrL2**. These results agree with the ROS generation abilities of **Ir1** and **Ir2** under 425 nm irradiation. The calculated energy level distributions of **IrL1** and **IrL2** are shown in Figure S30. The isoquinoline group in the C^N ligands in **IrL2** increased the electron delocalization compared to the pyridine ring in **IrL1**. The band gap of **IrL2** decreased, its absorption spectrum red-shifted (Figure S31), and its exciton transition ability significantly increased. Meanwhile, combined with the calculated singlet and triplet excitation energies of the Ir complexes at the TD-B3LYP/6-31G (d) SMD (solvent = H_2_O) level (Figure 2i), it was found that the singlet triplet energy level degeneracy of **IrL2** was significantly enhanced, especially in the lower triplet states, where the increase in degenerate orbitals contributes to the ISC ability of S_1_ and triplet orbitals. This is more conducive to the generation of ROS in **IrL2**, consistent with the experimental results. In **IrL2**, luminol is activated to the excited state with H_2_O_2_ and Hb, then, energy is transferred to the **Ir2** unit through CRET, and finally, the type I or II ROS are generated through electron transfer or energy transfer of the excited state of the PSs (Scheme 1). The above results demonstrate that **IrL2** has properties for type I and II ROS generation without an external light source.

### Preparation and Characterization of IrL2H NPs

Based on the above discussion, catalysis by Hb is needed for the outstanding CL-induced ROS production ability of **IrL2**. To improve the water solubility of **IrL2** while incorporating Hb into the NPs to release them into cancer cells together, liposome **IrL2H** NPs were prepared by combining **IrL2** with Hb (see the Experimental Section below; **IrL2** in a hydrophobic layer and Hb in a hydrophilic layer). The absorption, emission, and Fourier transform infrared spectra demonstrate the successful preparation of **IrL2H** (Figures 2j and S32). The morphology of **IrL2H** NPs was evaluated by transmission electron microscopy (TEM) to be spherical in shape (Figure 2k). The hydrodynamic size and polydispersity index (PDI) of **IrL2H** NPs were measured by dynamic light scattering (DLS), as shown in Figures 2k and 2l. The size of **IrL2H** was 153.8 nm, and there was no significant change in size within 5 days, implying that its stability in water basically satisfied the prerequisite for in vitro and in vivo applications.

### Intracellular CL and Uptake of IrL2H

The in vitro application of **IrL2H** was investigated with breast cancer 4T1 mice cells as a standard and readily available model cell line. The CL of **IrL2H** within cells was studied by adding different concentrations of **IrL2H** into 96-well plates with incubating 4T1 cells (Figure S34). At a concentration of 30 μg/mL, weak CL was observed, while at 60 μg/mL, the CL of **IrL2H** was more pronounced, indicating its good CL ability. The cellular uptake of **IrL2H** was tested by confocal laser scanning microscopy (CLSM) (Figure S35). After coincubation with 4T1 cells for 10 min, red luminescence of **IrL2H** was observed, indicating excellent cellular uptake of **IrL2H**. Subsequently, as the incubation time increased, red luminescence could be observed within 3–12 h, and the intensity was not weakened, indicating that the material could accumulate in cells. These results are prerequisite for its application in subsequent experiments.

### In Vitro CL-Excited PDT

**Figure 3:**
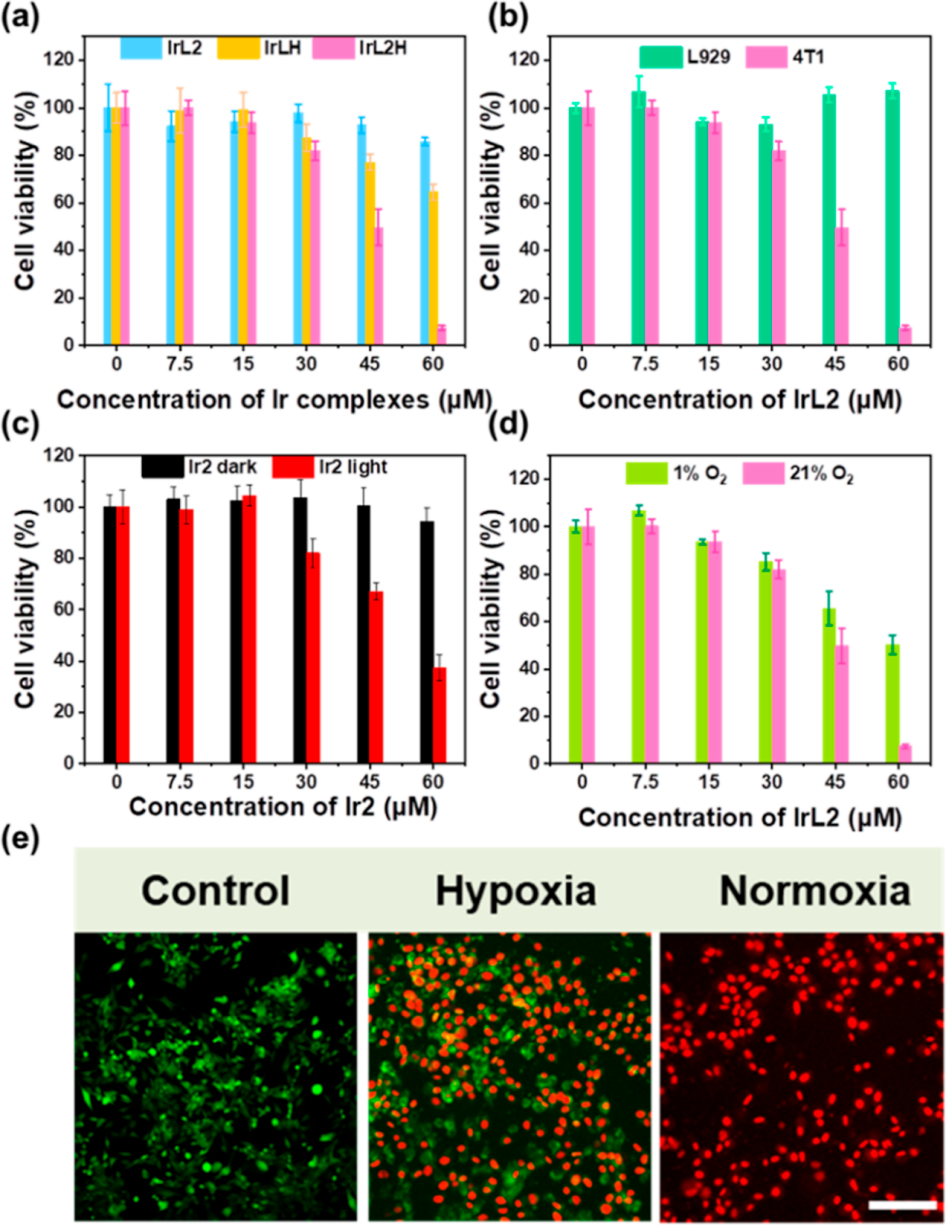
Relative viability of (a) 4T1 cells after 24 h coincubation with **IrL2**, **IrLH**, and **IrL2H** (0–60 μM); (b) 4T1 and L929 cells after 24 h coincubation with **IrL2H** (60 μM); (c) 4T1 cells with **Ir2** (0–60 μM) in darkness and under 425 nm light irradiation, and (d) 4T1 cells with **IrL2H** (0–60 μM) at different contents of O_2_. (e) Confocal fluorescence images of 4T1 cells costained with calcein-AM (live cells, green fluorescence) and PI (dead cells, red fluorescence) after treatment with **IrL2H** under normoxic and hypoxic conditions. Scale bar = 100 μm.

The viability of 4T1 cells was evaluated by using an MTT assay under various treatments in vitro. Figure 3a shows that the viability of the 4T1 cells is <20% in 60 μM **IrL2H**, while >80% of the 4T1 cells were viable in **IrL2** and >60% of 4T1 cells are viable in **IrLH** NPs. These results clearly demonstrate the superior PDT effect of **IrL2H**. To assess the effect of **IrL2H** on normal tissue cells, the viability of mouse fibroblasts L929 cells was studied by an MTT assay at different concentrations of **IrL2H** (Figure 3b). The results indicate that the toxicity of **IrL2H** to L929 cells is negligible, demonstrating its good biocompatibility. Furthermore, to assess the effect of different components in **IrL2H** on 4T1 cells, Hb and luminol + Hb were tested; almost no toxicity was observed (Figure S37). The cytotoxicity of **Ir2** under dark and light conditions showed that **Ir2** had almost no cytotoxicity under dark conditions, whereas a high level of phototoxicity was observed under 425 nm light, indicating that the cytotoxicity of **IrL2H** most likely originates from the excited **Ir2** unit (Figure 3c). The above results illustrate that **IrL2H** exhibits excellent cytotoxicity to 4T1 cells at 60 μM, which is attributed to the PDT effect of the **Ir2** unit irradiated by CRET, and **IrL2H** has almost no toxicity to normal cells, demonstrating its good biosafety.

The cytotoxicity of **IrL2H** in hypoxic cancer cells was assessed. As shown in Figure 3d, the viability of 4T1 cells decreases to <50% even in 1% O_2_ conditions with 60 μM **IrL2H** treatment. The IC_50_ values of **IrL2H** in 4T1 cells under normoxic and hypoxic conditions were calculated to be 36.89 and 80.91 μM, respectively. Live/dead cell staining (Figure 3e) shows that only red fluorescence could be observed in the normoxic environment compared with the control group, indicating the death of 4T1 cells after being treated with **IrL2H**. Both green and red fluorescence were observed after 4T1 cells were treated with **IrL2H**, suggesting that **IrL2H** can still partially kill cells under hypoxic conditions, which is consistent with the results of the MTT assay. Therefore, **IrL2H** is phototoxic through CL excitation in the hypoxic TME.

**Figure 4:**
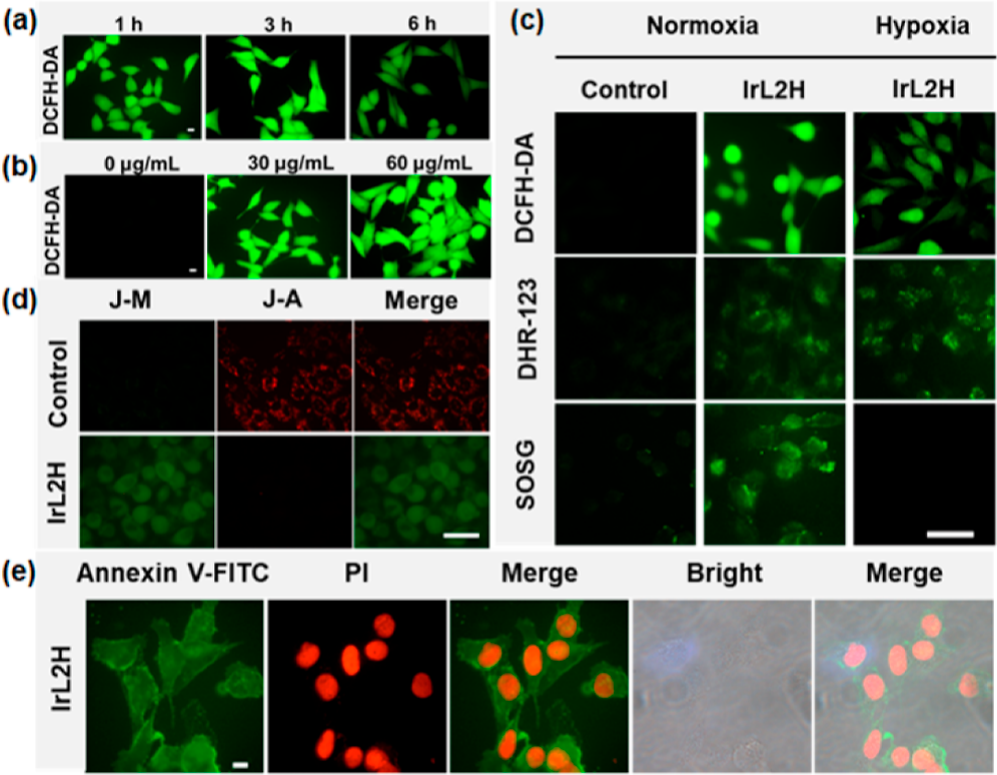
Confocal fluorescence images for the detection of ROS after treatment with (a) **IrL2H** for different times and (b) different concentrations of **IrL2H**. Scale bar = 10 μm. (c) Confocal fluorescence images for detecting the different types of ROS after treatment with **IrL2H**. Scale bar = 50 μm. (d) Confocal fluorescence imaging of MMP in 4T1 cells incubated with **IrL2H** via a subsequent JC-1 dye assay. Scale bar = 50 μm. (e) Death of 4T1 cells induced by **IrL2H** and staining with dual fluorescence of Annexin V-FITC/PI. Scale bar = 10 μm.

To better understand the PDT mechanism, the ROS production of **IrL2H** under different conditions was tested by using DCFH-DA as an indicator after different incubation times. As shown in Figure 4a, the intracellular green fluorescence increased within 1–3 h of incubation with **IrL2H** and then weakened by 6 h, indicating an initial increase in the ROS level and then a decrease, maybe due to the decay of the CL. Figure 4b shows that the green fluorescence increased as the **IrL2H** concentration increased, implying that the intracellular ROS level increased with the enhancement of the **IrL2H** concentration. The type of ROS generated by **IrL2H** in normoxic and hypoxic 4T1 cells was investigated by using DHR-123 and Singlet Oxygen Sensor Green (SOSG) as indicators for type I and II ROS, respectively. As shown in Figure 4c, the green fluorescence of DHR-123 and SOSG was observed under normal oxygen conditions, demonstrating the good ability of **IrL2H** to produce type I and type II ROS. However, under hypoxic conditions, only the fluorescence of DHR-123 was observed, indicating that **IrL2H** can generate type I ROS under hypoxic conditions but not type II ROS due to insufficient O_2_ to provide energy transfer substrates. In summary, the intracellular ROS production of **IrL2H** increases with increasing **IrL2H** concentration, and **IrL2H** could still perform PDT in hypoxic cells through a type I process.

Apoptosis is the most common PDT cell death pathway.^68^ Mitochondrial membrane potential (MMP) is a parameter that reflects the level of cellular health.^68^ The loss of MMP may stimulate apoptosis. A JC-1 dye assay was applied in 4T1 cells to evaluate the damage of the MMP (Figure 4d). Strong red fluorescence from aggregated-JC-1 was observed in the control group, while the group treated with **IrL2H** showed a green fluorescence from free-JC-1, indicating that **IrL2H** induced a loss of MMP. Annexin V-FITC/PI dual fluorescence staining experiments were performed using CLSM to detect the effect of **IrL2H** on 4T1 cells. Annexin V-FITC emits green fluorescence, while PI emits red fluorescence. As shown in Figure 4e, cells treated with **IrL2H** show strong green and red fluorescence, indicating the occurrence of apoptosis. The above in vitro results, with excellent cytotoxicity of **IrL2H** and its CL-triggered PDT-induced cell apoptosis, laid the foundation for in vivo experiments.

### In Vivo CL Imaging and PDT

**Figure 5:**
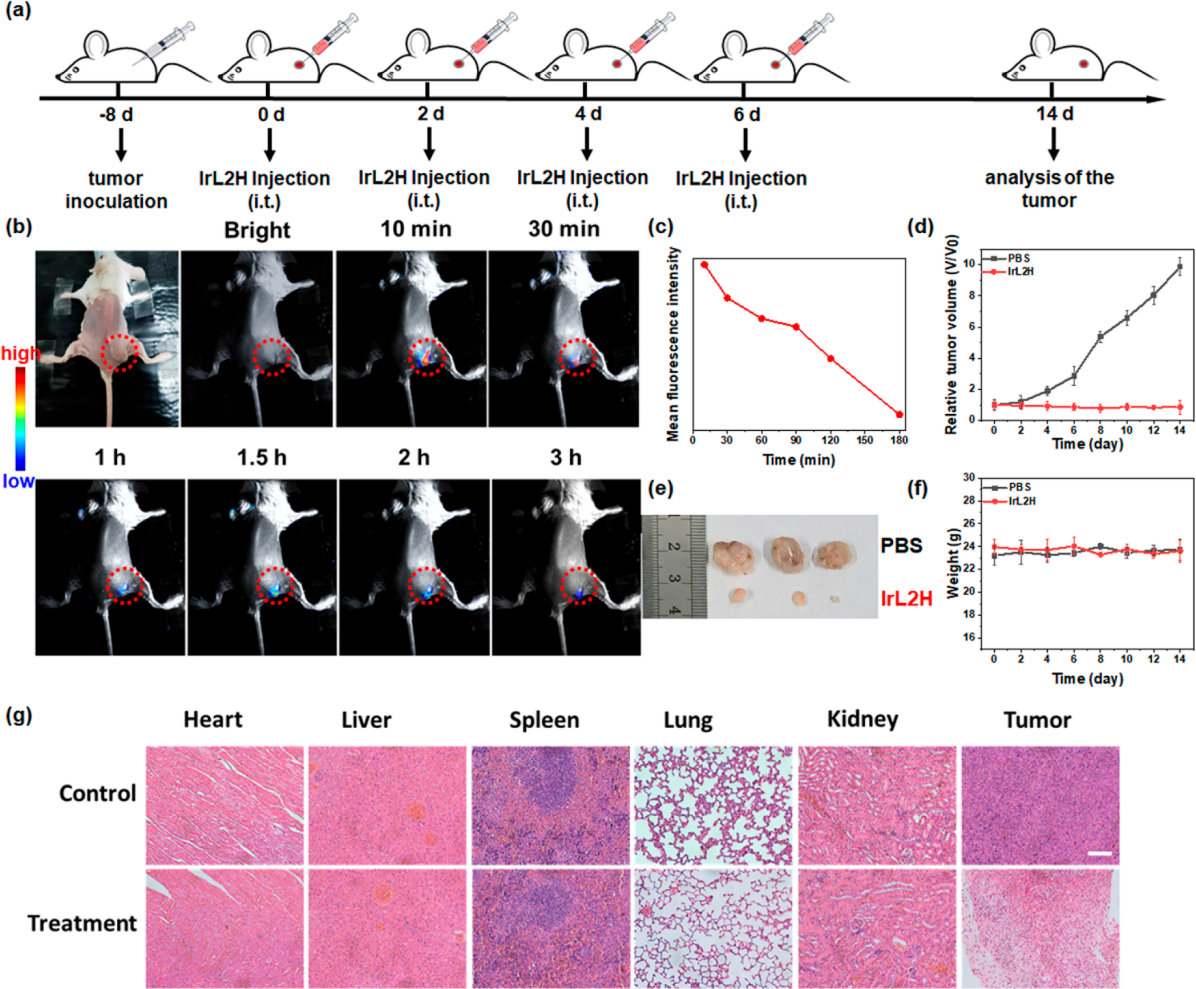
**IrL2H**-mediated inhibition of tumor growth in the 4T1 tumor model. (a) Timeline of the treatment. (b) Imaging of **IrL2H** in tumors; and (c) curve of the average fluorescence intensity at the tumor site over time. (d) Relative tumor volume in different groups. (e) Photos of tumors after treatment. (f) Changes in the body weights of mice. (g) H&E staining images of tumor tissues from the different groups on day 14; scale bar: 100 μm.

Inspired by the excellent in vitro anticancer efficiency of **IrL2H**, in vivo experiments were undertaken in mice bearing 4T1 tumors. The CL images of **IrL2H** in the tumors were obtained. As shown in Figure 5b,c, a tumor model was constructed by injecting 4T1 cells into the right thigh of mice, and **IrL2H** and H_2_O_2_ were intratumorally injected to detect their CL image. The CL intensity of **IrL2H** was bright after injection, then slightly decreased between 10 and 30 min, then weakened more rapidly after 1.5 h, and finally disappeared after 3 h. The results indicate that **IrL2H** has a promising in vivo CL imaging performance.

We further applied **IrL2H** to in vivo therapy. As shown in Figure 5a, a mouse model was established by subcutaneous injection of 4T1 cells into the left thigh. Then, tumor-bearing mice were randomly divided into two groups, each consisting of 4 mice: (i) PBS and (ii) **IrL2H**. Intratumoral injection therapy was performed on days 0, 2, 4, and 6, and then, each group of mice was observed for 14 days. The changes in tumor volume and weight of each mouse were recorded (Figure 5d–f). The relative tumor volume of the PBS group increased about 10 times after 14 days. In contrast, there was no significant change in tumor volume in the **IrL2H** group. After 14 days of treatment, the mice were sacrificed, and the tumors, as well as the subject organs (heart, liver, spleen, lung, and kidneys), were excised for histological analysis. As shown from hematoxylin and eosin (H&E) staining (Figure 5g), there were many stromal tight cells and intact nuclei and cytoplasm in the control group, indicating the good condition of the cells. The tissue space presented a hiatus, and the tumor cells were destroyed in the **IrL2H** group, indicating that **IrL2H** has a good inhibitory effect on tumor growth. Within 14 days, there was no significant change in the weight of the mice, and histological analysis of the main organs after 14 days of treatment showed almost no pathological changes (Figure 5g), indicating that **IrL2H** imparts almost no damage to normal tissues, demonstrating its good biocompatibility. The above results indicate that **IrL2H** has an excellent ability to inhibit tumor growth through self-CL-induced PDT, verifying its potential application in cancer treatment.

## Conclusions

In conclusion, Ir complex **Ir2** was rationally synthesized by extending the π-conjugation of the N ligand to give a good overlap between the absorption spectrum of the complex and the emission of luminol. Luminol was covalently attached to the auxiliary ligand of **Ir2** through amide condensation reactions to construct a chemiluminescent molecule **IrL2**. The experimental results show that the CL of **IrL2** increases with an increase of H_2_O_2_ and Hb concentrations, and **IrL2** still exhibits excellent CL properties at pH = 6.5. **IrL2** generates types I and II ROS excited by CL under the catalysis of H_2_O_2_ and Hb. Liposome **IrL2H** NPs, constructed with **IrL2** and Hb, exhibited good stability in aqueous solutions. In vitro experiments showed that **IrL2H** is effectively internalized by cells, producing CL in cells, and is nontoxic to normal cells. The CRET from luminol to the **Ir2** unit excited the Ir complex to generate type I and type II ROS, leading to apoptosis of 4T1 cells. In vivo experiments demonstrate that **IrL2H** images tumors by self-CL and inhibits tumor growth following intratumoral injection. This work provides a versatile precedent for using functionalized transition metal complexes to self-generate CL for PDT, thereby extending the molecular platform for tackling the enduring problems of an external light source and the hypoxic tumor environment in clinical applications.

## Experimental Section

### Materials and Instruments

Materials for organic synthesis were purchased from the Energy Chemical Company. ICG, DMPO, TEMP, and DHR 123 were purchased from Energy Chemical Company. RPMI medium 1640 was purchased from the Solarbio Life Science Company. Fetal bovine serum (FBS) was purchased from Sigma-Aldrich. 3-(4,5-Dimethyl-2-thiazolyl)-2,5-diphenyl-2*H*-tetrazolium bromide (MTT), 2,7-dichlorofluorescencein diacetate (DCFH-DA), and the cell viability (live dead cell staining) assay kit were purchased from Shanghai Beyotime Biotechnology Co., Ltd.

NMR spectra were recorded at 25 °C on a Varian 500 MHz spectrometer. HPLC used a Shimadzu LC-16A liquid chromatograph. UV–vis absorption spectra were recorded on a Shimadzu UV-3100 spectrophotometer. The photoluminescence spectra, excited state lifetimes (τ), and photoluminescence quantum yields (Φ) were recorded on an Edinburgh FLS920 spectrofluorometer under air at room temperature. TEM images of the samples were taken with a TECNAI F20 microscope. Diameter and diameter distributions of the NPs were determined by a Malvern Zetasizer Nano instrument for DLS. CLSM images were taken using a ZeissLSM 700 instrument (Zurich, Switzerland). The hypoxic conditions were achieved using a biological hypoxic incubator (MIC-101 from Billups-Rothenberg Company).

### Synthesis

The synthesis of **Ir1** and **Ir2** followed standard procedures, as reported in the Supporting Information. The synthetic route to [Ir(C^N)_2_(N^N)]^+^ complexes leads to a racemic mixture of Δ and Λ stereoisomers, which (as in the present case) are generally not separated.^69^

### Complexes **IrL1** and **IrL2**

**Ir1** (74.5 mg, 0.1 mmol) [or **Ir2** 84.5 mg (0.1 mmol)] and luminol (42.5 mg, 0.24 mmol) were dissolved into dry DMF (3 mL) and stirred for 30 min at 0 °C. Then, *N*,*N*-diispropylethylamine (DIPEA) (38.78 mg, 0.3 mmol) was added into the mixture. Subsequently, 1-hydroxybenzotriazole (HOBt) (37 mg, 0.3 mmol) and 1-ethyl-3-(3-(dimethylamino)propyl)carbodiimide (EDCl) (57.5 mg, 0.3 mmol) were added to the mixture and stirred for 1 h in 0 °C. After that, the mixture was stirred under N_2_ at room temperature for 3 days. Cold water was added to terminate the reaction, and the product was extracted with dichloromethane; the organic layer was collected, and the solvent was evaporated to obtain the crude product as a red solid. The product was purified by silica gel column chromatography with CH_2_Cl_2_/MeOH (100:1–30:1 v/v) as the eluent.

**IrL1**: Yield: 29%. ^1^H NMR (500 MHz, DMSO-*d*_6_ δ [ppm]): 13.52–9.91 (m, 6H), 9.31 (s, 1H), 9.22 (s, 1H), 8.93 (d, *J* = 8.3 Hz, 1H), 8.20 (d, *J* = 7.6 Hz, 2H), 8.09–8.01 (m, 5H), 7.94–7.85 (m, 6H), 7.64 (dd, *J* = 16.3, 8.2 Hz, 4H), 7.07 (s, 2H), 6.97 (s, 2H), 6.85 (d, *J* = 5.0 Hz, 2H), 6.12–6.08 (m, 2H) (Figure S3). ^13^C NMR (151 MHz, DMSO-*d*_6_ δ [ppm]): 167.90, 167.53, 167.50, 165.03, 156.13, 151.64, 150.67, 148.98, 144.82, 141.69, 139.83, 139.05, 137.59, 133.03, 132.42, 129.65, 129.22, 125.82, 125.31, 124.29, 124.21, 123.80, 122.04, 120.68, 118.48 (Figure S4). ^19^F NMR (565 MHz, DMSO-*d*_6_ δ [ppm]): −69.42, −70.68 (Figure S5). MS:(MALDI-TOF) [*m*/*z*] 1064.2993 (M^+^) (Figure S6). Calcd for C_50_H_34_F_6_IrN_10_O_6_P: C 49.71, H 2.84, N 11.59; found, C 49.69, H 2.86, N 11.58.

**IrL2**: Yield: 24%. ^1^H NMR (500 MHz, DMSO-*d*_6_ δ [ppm]): 11.75 (s, 3H), 9.19 (d, *J* = 63.9 Hz, 1H), 9.02 (s, 1H), 8.71–8.57 (m, 1H), 8.40 (d, *J* = 4.4 Hz, 1H), 8.16–7.45 (m, 9H), 7.25–7.08 (m, 1H), 7.02–6.85 (m, 1H), 6.27–6.15 (m, 7.7 Hz, 1H) (Figure S7). ^13^C NMR (151 MHz, DMSO-*d*_6_ δ [ppm]): δ 168.61, 166.62, 166.43, 164.47, 156.25, 153.21, 153.05, 150.30, 144.83, 141.88, 141.84, 139.29, 137.32, 136.62, 133.03, 132.51, 132.19, 129.71, 128.86, 128.80, 128.43, 126.27, 125.89, 125.71, 124.29, 122.38, 122.20, 121.47, 118.89 (Figure S8). ^19^F NMR (565 MHz, DMSO-*d*_6_ δ [ppm]): −69.45, −70.71 (Figure S9). MS:(MALDI-TOF) (*m*/*z*) 1163.27 (M^+^) (Figure S10). Calcd for C_58_H_38_F_6_IrN_10_O_6_P: C 53.25, H, 2.93, N 10.71; found, C 53.26, H 2.93, N 10.68.

Note: The ^19^F NMR spectra of **IrL1 and IrL2** confirm that the PF_6_ anions are retained in these structures, as expected from literature precedents for similar amide bond-forming reactions.^70,71^

### Preparation of **IrL2H**

Lecithin (8.5 mg), cholesterol (2 mg), and DSPE-PEG52000 (1.5 mg) were mixed with **IrL2** (1.2 mg) and dissolved in chloroform (3 mL). The mixture was dried via a rotary evaporator under decreased pressure to form a red lipid film; **IrL2** could be in the hydrophobic layer due to its poor water solubility. Hb (2 mL) (1 mg/mL) was added into the lipid film, followed by shaking at room temperature for 30 min to encapsulate into the hydrophilic layer. The suspension was centrifuged at 10,000 rpm for 10 min and washed with ultrapure water to remove the unencapsulated Hb. Finally, the liposomes were dispersed in a PBS buffer to form **IrL2H**. The loading content of **IrL2** in **IrL2H** was determined to be 5.06 wt % and the loading content of Hb in **IrL2H** was determined to be 2.32 wt %, according to their standard curves (Figures S13 and S14). The control groups were prepared by a similar procedure.

### CL Response of **IrL1** and **IrL2** Dots in Various Conditions

100 μM **IrL1** or **IrL2**, 200 μM H_2_O_2_, and 10 μg mL^–1^ Hb were mixed in a quartz cell at pH = 6.5 to detect the CL spectra of **IrL1** and **IrL2**. Solvent: DMF/H_2_O = 1/1000, v/v. (i) **IrL2** (50 μg mL^–1^) was mixed with H_2_O_2_ at different concentrations (0–400 μM) and Hb (5 μg mL^–1^) at pH = 7.4 in a black 96-well plate. (ii) **IrL2** at different concentrations (0–100 μg mL^–1^) was mixed with H_2_O_2_ (200 μM) and Hb (5 μg mL^–1^) at pH = 7.4 in a black 96-well plate. (iii) **IrL2** (50 μg mL^–1^) was mixed with H_2_O_2_ (200 μM) and Hb (5 μg mL^–1^) at different pH values (5.0, 5.5, 6.5, 7.4, and 8.0) in a black 96-well plate. (iv) **IrL2** (50 μg mL^–1^) was mixed with H_2_O_2_ (200 μM) and Hb at different concentrations (0–20 μg mL^–1^) at pH = 7.4 in a black 96-well plate. (v) **IrL1** and **IrL2** (50 μg mL^–1^) was mixed with H_2_O_2_ (200 μM) and Hb (5 μg mL^–1^) at pH = 7.4 in a black 96-well plate. Luminescence images (emission filter, long pass, 510 nm; exposure time, 10 min) were acquired with a TUCSEN FL 9BW image system.

### Test Method for ROS Generation in Solution

EPR analysis was performed using TEMP or DMPO as the spin-trap agent. To TEMP (10 μL) [or MeOH solution containing 20 mM DMPO (30 μL)] was added 20 μM **Ir1** and **Ir2**. The spectra were monitored in a range of 3380–3460 G after the solution was irradiated by a 425 nm LED at 20 mW cm^–2^ for 5 min. To TEMP (10 μL) was added (i) 20 μM **IrL1** and **IrL2**, 300 μM H_2_O_2_, and 1 μg mL^–1^ Hb; (ii) 20 μM **IrL1** and **IrL2** and 300 μM H_2_O_2_; (iii) 300 μM H_2_O_2_ and 1 μg mL^–1^ Hb; or (iv) 20 μM **IrL1** and **IrL2**. Solvent: DMF/H_2_O = 1/1000, v/v, pH = 6.5. The spectra were monitored in a range of 3380–3460 G. Microwave power: 5.012 mW.

### ^1^O_2_ Quantum Yield Measurements

The ^1^O_2_ quantum yield of aggregation-induced emission NPs in water (Φ) upon LED irradiation (425 nm, 20 mW cm^–2^) was determined using ICG as an indicator and using Rose Bengal (RB) as the standard; ref (72) The absorbance decrease of ICG at 790 nm was recorded for different durations of light irradiation to obtain the decay rate of the photosensitizing process. The ROS yield was calculated by using the following equation:
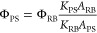
where *K*_PS_ and *K*_RB_ are the decomposition rate constants of the photosensitizing process determined by the plot ln(*A*_0_/*A*) versus irradiation time (Figures S21 and S22). *A*_0_ is the initial absorbance of ICG, while *A* is the absorbance of ICG after different irradiation times. *A*_PS_ and *A*_RB_ represent the light absorbed by Ir complex PSs and RB at 425 nm, respectively. Φ_RB_ is the ^1^O_2_ quantum yield of RB, which is 0.75 in water.

### Calculation Methods

All calculations were completed in the Gaussian 16 software package. The Hay Wadt effective nuclear potential (ECP) and double ξ optimize the structure of the base set LANL2DZ to obtain a stable structure without imaginary frequencies. FMO orbital information on Ir complex molecules, including energy levels and distributions, was obtained at the same theoretical level. Excited state calculations were conducted in water at the levels of TD-B3LYP/6-31Gd, SMD, solvent = H_2_O, and the results were used to analyze the optoelectronic properties of the material.

### Cell Culture Methods

4T1 cells were cultured in this experiment. The culture medium was prepared with RPMI medium 1640 containing 10% (v/v) FBS. The cell culture flask was placed in an incubator under the following conditions: normoxic conditions: cells were maintained in a humidified atmosphere containing 5% CO_2_ of 37 °C; hypoxic conditions: cells were maintained at 37 °C in a humidified atmosphere containing 1% O_2_, 5% CO_2_, and 94% N_2_. L929 cells were cultured in this experiment. The culture medium was prepared with Dulbecco’s modified Eagle medium (DMEM) containing 10% (v/v) FBS. The cell culture flask was placed in an incubator in an atmosphere containing 5% CO_2_ at 37 °C.

### Cytotoxicity Test Methods

4T1 cells were seeded in 96-well plates at a density of 10,000 cells per well and cultured in the incubator under different conditions (normoxia or hypoxia) for 24 h. After aspirating the old culture medium, 100 μL of RPMI medium 1640 containing different concentration gradient PSs (0–60 μM) were added to each well, and the cells were placed in the incubator for 24 h. After incubation, the cells were placed in an incubator for 24 h. Then, the viability of the cells was detected by an MTT assay.

### Intracellular Uptake

4T1 cells were seeded in confocal culture dishes at a density of 50,000 cells per well. RPMI medium 1640 containing NPs was incubated for different times. After incubation, the cells were fixed and then stained with 4′,6-diamidino-2-phenylindole (DAPI). The uptake of **IrL2H** by cells was observed by CLSM.

### Evaluation of Intracellular ROS Production Capacity

4T1 cells were seeded in confocal dishes at a density of 50,000 cells per well. Different formulations (control, **IrL2H**) were added to the medium, and the cells were incubated for another 24 h. For the ROS level test, DCFH-DA (1 μL) [or SOSG (1 μL) or DHR-123 (3 μL)] was dissolved in blank RPMI medium 1640 without FBS and added to the confocal culture dish. After treatment in the dark for 20 min, the medium containing indicator was aspirated, washed twice with PBS, and 1 mL of PBS was added, followed by CLSM analysis. To observe the green fluorescence intensity in the cells, λ_ex_ = 465–495 nm and λ_em_ = 415–555 nm for indicators.

### Live/Dead Staining Test Methods

4T1 cells were seeded in confocal dishes at a density of 50,000 cells per well. Different formulations (control, **IrL2H**) were added to the medium, and the cells were incubated for another 24 h. Detection buffer containing Calcein-AM and PI was added to the confocal dishes. The fluorescence image was observed under an inverted fluorescence microscope to assess the cell survival state: λ_ex_ = 540–580 nm, λ_em_ = 600–660 nm for PI and λ_ex_ = 465–495 nm, λ_em_ = 415–555 nm for Calcein-AM.

### CL Response of IrL2H in 4T1 Cells

4T1 cells were incubated with **IrL2H** at different concentrations in a 96-well plate. Luminescence images (emission filter, long pass 510 nm; exposure time, 10 min) were acquired with a TUCSEN FL 9BW image system.

### Detection of Mitochondrial Membrane Potential

The 4T1 cells were incubated in a glass-bottom dish at a density of 50,000 cells per dish for 24 h, and the,n the cells were subjected to different treatments (Control, **IrL2H**). After 3 h, the culture medium was replaced. Next, the cells were incubated with JC-1 dye (5 μg/mL) at 37 °C for 20 min. Then, the cells were washed three times with PBS. After that, they were imaged by CLSM. λ_ex_ = 540–580 nm, λ_em_ = 600–660 nm for J-A and λ_ex_ = 465–495 nm, λ_em_ = 415–555 nm for J-M.

### Annexin V-FTIC/PI Assays

4T1 cells were seeded in confocal dishes at a density of 50,000 cells per well. Different formulations (control, **IrL2H**) were added to the medium, and the cells were incubated for another 24 h. Finally, the cells were stained with Annexin-FITC/PI for 15 min and then were imaged by CLSM. λ_ex_ = 540–580 nm, λ_em_ = 600–660 nm for PI and λ_ex_ = 465–495 nm, λ_em_ = 415–555 nm for Annexin V-FTIC.

### Animals and Tumor Model

The mice experiments were performed in accordance with animal regulations and management protocols. All animal experiments were approved by the Institutional Animal Care and Use Committee of Northeast Normal University. BALB/c mice (female, 6–8 weeks) were purchased from the Vital River Company in Beijing. 4T1 cells (1.0 × 10^6^/tumor, 100 μL in PBS) were subcutaneously injected into the flank of each mouse to establish the 4T1 tumor model.

### In Vivo CL Imaging

To study the change of CL of **IrL2H** in vivo, BALB/c mice anesthetized were intratumorally injected with **IrL2H** solution (100 μg mL^–1^) and 5 μL of H_2_O_2_ (1 mM) into the same field. The CL images were captured post injection for different times. The CL images were taken immediately by a TUCSEN FL 9BW image system (emission filter, long pass 510 nm; exposure time 20 min). Following a literature precedent, intratumoral injection can maximize the accumulation of radiosensitizers within a tumor compared to intravenous injection and further enhance efficacy.^73^ We also attempted in vivo imaging with intravenous injection, and almost no luminescence was observed under the same detection conditions as those under intratumoral injection. Therefore, intratumoral injection was used to accumulate more NPs to improve the therapeutic effect in our system.

### Evaluation of Antitumor Efficacy In Vivo

When the tumor volume grew to about 60 mm^3^, the mice were randomly separated into two groups with 4 mice in each group. Then, the mice were injected with PBS (i) and **IrL2H** (12 mg/kg) (ii). The tumor sizes and body weights of the mice were monitored every 2 days during the 14 days of treatment. The tumor volume was measured as volume = (*L* × *W*^2^/2), where *L* (length) and *W* (width) are the two tumor dimensions. On day 14, the mice in each group were euthanized; the tumors and major organs were harvested for subsequent analysis. The tissues were fixed with formalin and then paraffin-embedded. Furthermore, the paraffin-embedded tissues were sectioned and stained with H&E to determine tissue damage after observation under a microscope.

## Data Availability

The data associated with this article are available in the manuscript and Supporting Information files. Additional data will be made available on request.

## Supporting Information Available

Material structure data; detailed ROS detection spectra; control group NP data; additional calculation results; detailed cell uptake; and in vitro experimental results of control groups (PDF)

## Abbreviations

CL: chemiluminescence
CRET: chemiluminescence resonance energy transfer
LED: light emitting diode
PDT: photodynamic therapy
ROS: reactive oxygen species
TME: tumor microenvironment
Hb: hemoglobin
NPs: nanoparticles
PS: photosensitizer.
