# Supplementary material for: Self-Chemiluminescence-Triggered Ir(III) Complex Photosensitizer for Photodynamic Therapy against Hypoxic Tumor
Source: Inorg Chem. 2024 Aug 16;63(35):16404–17. doi: 10.1021/acs.inorgchem.4c02399 (PMC11372751; doi:10.1021/acs.inorgchem.4c02399)
Supplement: Supplementary file 1 — ic4c02399_si_001.pdf [file ic4c02399_si_001.pdf]

Supporting Information (SI)

Self-chemiluminescence Triggered Ir(III) Complex Photosensitizer for Photodynamic Therapy against Hypoxic Tumor

Shengnan Liu [a], *Haoran Chen* [b], *Qi Wu* [a], *Yan Sun* [a], *Yu Pei* [a], *Ziwei Wang* [a], *Dongxia Zhu** [a], *Gungzhe Li** [c], *Martin R. Bryce** [d] *and Yulei Chang** [b]

[a] Dr. S. Liu, Dr. Q. Wu, Dr. Y. Sun, Dr. Y. Pei, Dr. Z. Wang and Prof. D. Zhu

Key Laboratory of Nanobiosensing and Nanobioanalysis at Universities of Jilin Province, Department of Chemistry, Northeast Normal University, 5268 Renmin Street, Changchun, Jilin Province 130024, P.R. China.

*E-mail: zhudx047@nenu.edu.cn

[b] Dr. H. Chen, Prof. Y. Chang

State Key Laboratory of Luminescence and Applications, Changchun Institute of Optics, Fine Mechanics and Physics, Chinese Academy of Sciences, Changchun Jilin Province 130033, P.R. China.

*E-mail: yuleichang@ciomp.ac.cn

[c] Prof. G. Li

Jilin Provincial Science and Technology Innovation Center of Health Food of Chinese Medicine, Changchun University of Chinese Medicine, Changchun, Jilin Province 130117, P. R. China.

E-mail: 1993008106@qq.com

[d] Prof. Martin R. Bryce

Department of Chemistry, Durham University, Durham, DH1 3LE, UK.

*E-mail: m.r.bryce@durham.ac.uk

Table of Contents

1. Experimental Section **S3**

2. Supporting Figures S1-S39 **S5**

3. Supporting Table S1 **S25**

Experimental Section

Synthesis of the dichloro-bridged diiridium complexes [Ir(ppy)$_2$Cl]$_2$ and [Ir(pqy)$_2$Cl]$_2$

2-Phenylpyridine (ppy) (466 mg, 3 mmol) [or 1-phenylisoquinoline (pqy) (616 mg, 3 mmol)] and iridium trichloride hydrate (352 mg, 1 mmol) were dissolved in a mixture of 2-ethoxyethanol (30 mL) and water (10 mL) and reacted at 120 ^{o}C under an inert atmosphere of N_2 for 24 h. After cooling to room temperature, water was added to the solution and the precipitate was collected by filtration. The precipitate was washed with water and EtOH and dried at 68 ^{o}C to give the dichloro-bridged diiridium complex [Ir(ppy)$_2$Cl]$_2$ or [Ir(pqy)$_2$Cl]$_2$ which was used directly in the subsequent reaction.

Synthesis of complexes Ir1 and Ir2

A yellow suspension of the dichloro-bridged diiridium complex [Ir(ppy)$_2$Cl]$_2$ (1.072 g, 1 mmol) or [Ir(pqy)$_2$Cl]$_2$ 1.272 g (1 mmol) and the N^N bridging ligand (490 mg, 2 mmol) in MeOH (50 mL) and CH_2Cl_2 (50 mL was refluxed under an inert atmosphere of N_2 in the dark for 12 h. The red solution was then cooled to room temperature, and solid ammonium hexafluorophosphate (0.37 g, 20 mmol) was added to the solution. The mixture was stirred for 30 min at room temperature and the suspension was then filtered and the precipitate was washed with petroleum ether and dried to obtain the product.

Ir1: light red solid. Yield: 89%. ^{1}H NMR (500 MHz, DMSO-*d$_6$* δ [ppm]): δ 9.20 (s, 1H), 8.28 (d, J = 8.1 Hz, 1H), 8.05 (d, J = 5.2 Hz, 1H), 7.99 (d, J = 5.6 Hz, 1H), 7.93 (d, J = 7.1 Hz, 2H), 7.65 (d, J = 5.5 Hz, 1H), 7.13 (t, J = 6.3 Hz, 1H), 7.03 (t, J = 7.6 Hz, 1H), 6.92 (t, J = 7.4 Hz, 1H), 6.17 (d, J = 7.6 Hz, 1H). (Figure S1).

Ir2: Orange red solid. Yield: 91%. ^{1}H NMR (500 MHz, DMSO-*d$_6$* δ [ppm]): δ 9.25 (s, 1H), 9.03 – 8.97 (m, 1H), 8.38 (d, J = 8.0 Hz, 1H), 8.09 (dd, J = 6.1, 3.5 Hz, 1H), 7.99

(dd, J = 5.6, 1.2 Hz, 1H), 7.93 – 7.85 (m, 3H), 7.55 (dd, J = 16.6, 6.5 Hz, 2H), 7.14 (t, J = 7.7 Hz, 1H), 6.92 (t, J = 7.4 Hz, 1H), 6.19 (d, J = 7.1 Hz, 1H). (Figure S2).

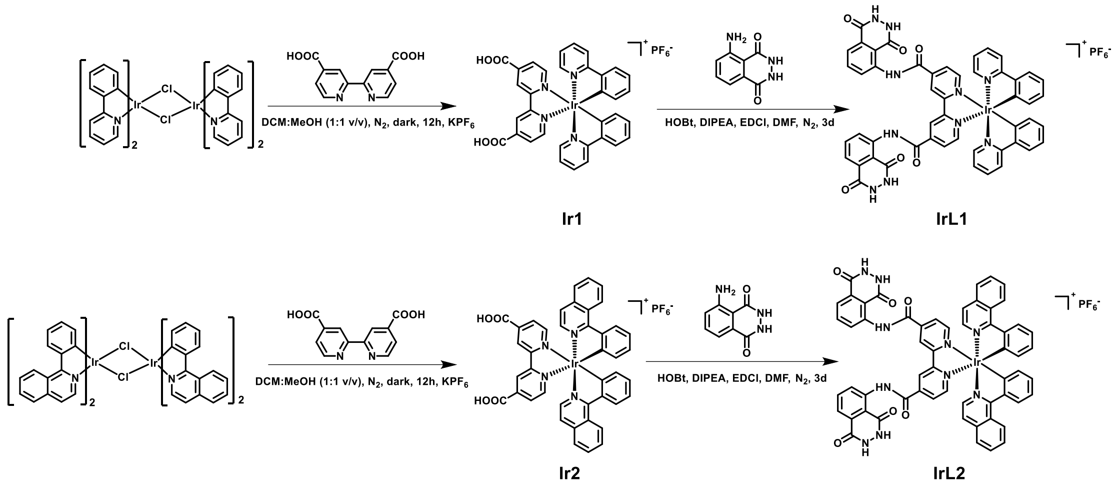

Scheme S1. Synthetic route for **Ir1**, **IrL1**, **Ir2** and **IrL2**.

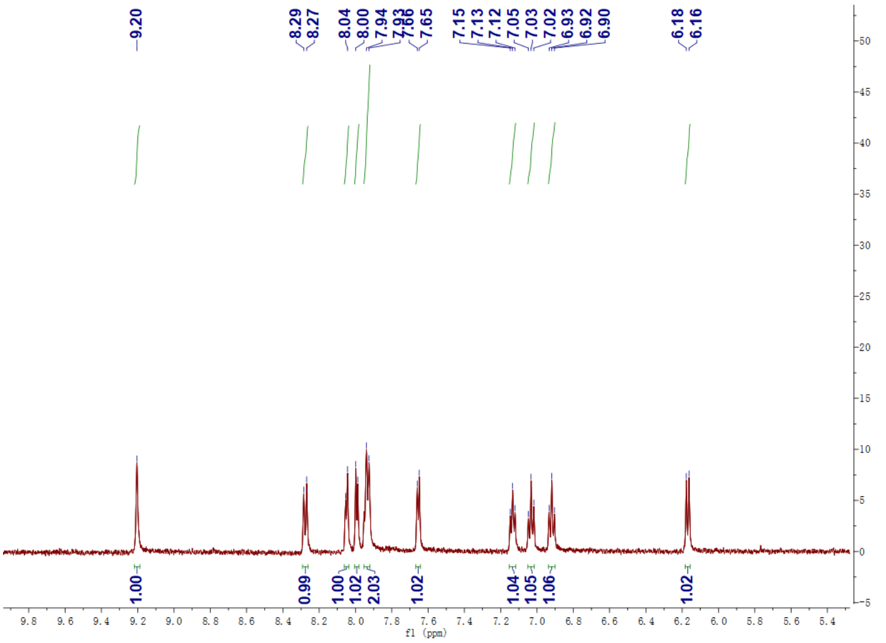

Figure S1 ^{1}H NMR spectrum of **Ir1** in DMSO-d_6 at room temperature.

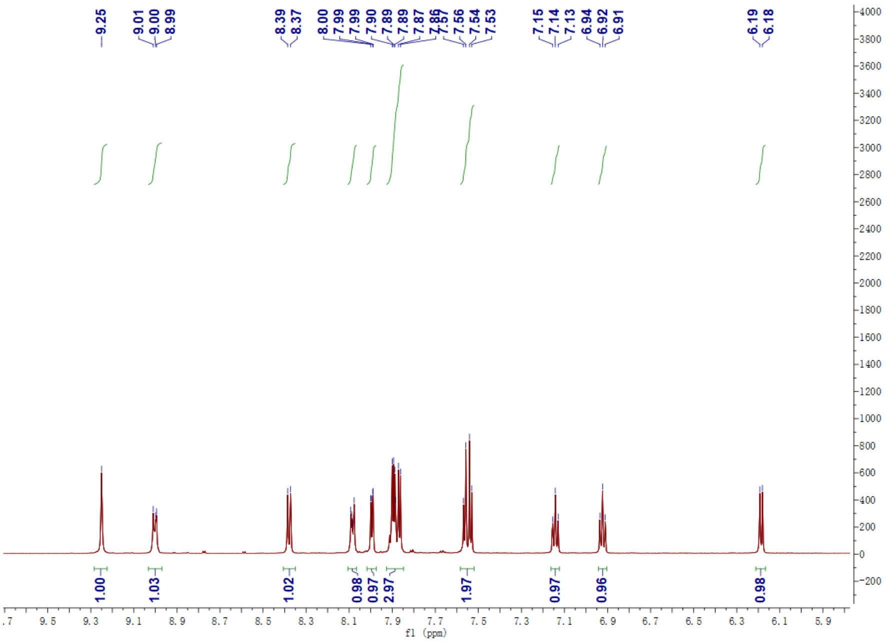

Figure S2 ^{1}H NMR spectrum of **Ir2** in DMSO-d_6 at room temperature.

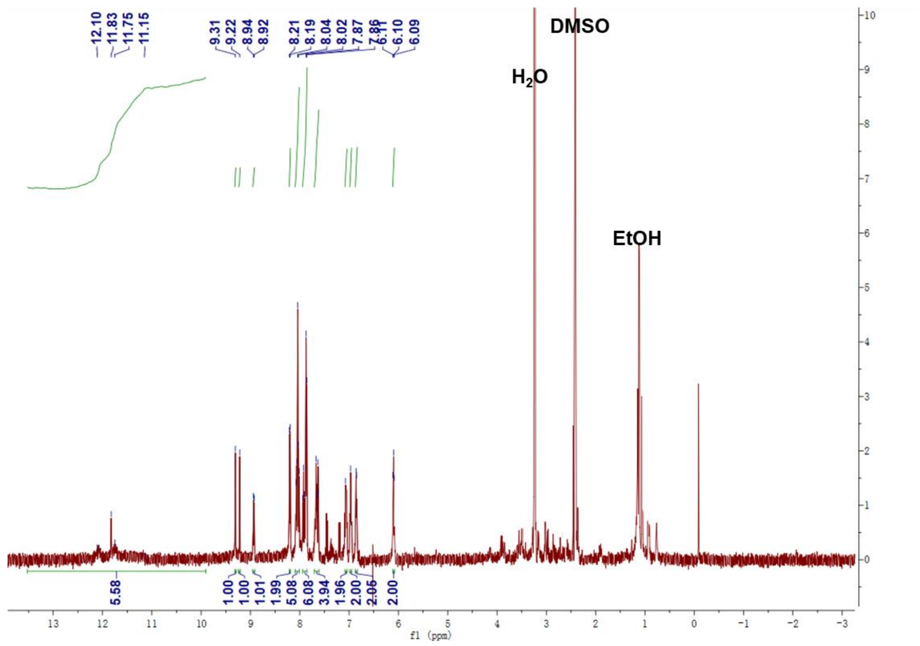

Figure S3 ^{1}H NMR spectrum of **IrL1** in DMSO-d_6 at room temperature.

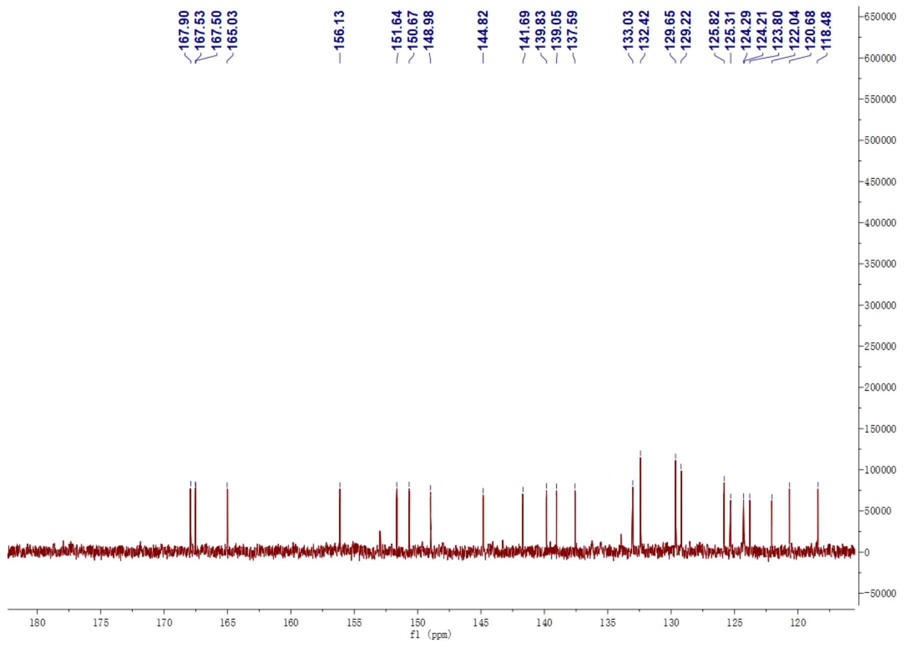

Figure S4 ^{13}C NMR spectrum of **IrL1** in DMSO-d_6 at room temperature.

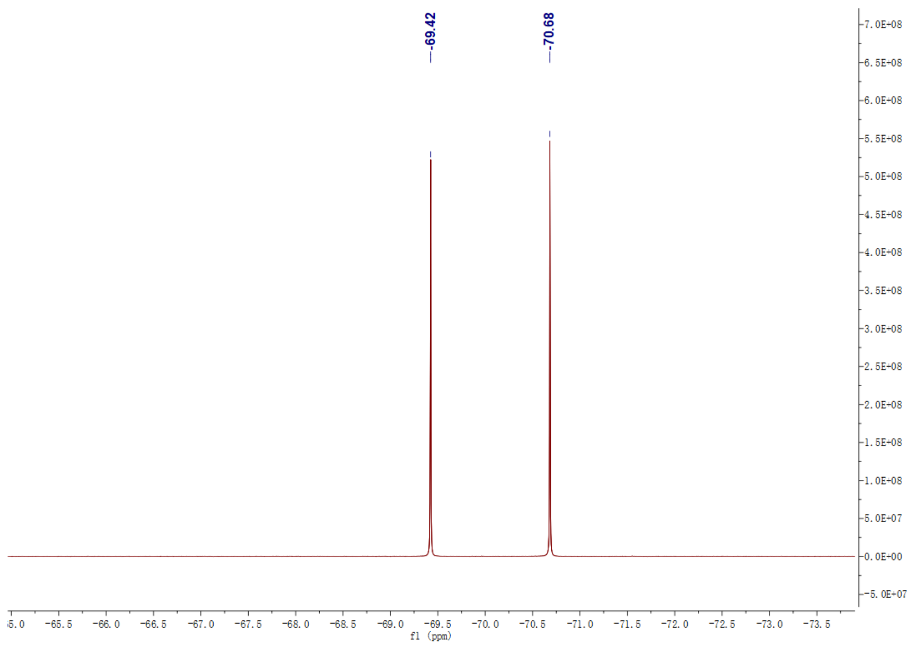

Figure S5 ^{19}F NMR spectrum of **IrL1** in DMSO-d_6 at room temperature.

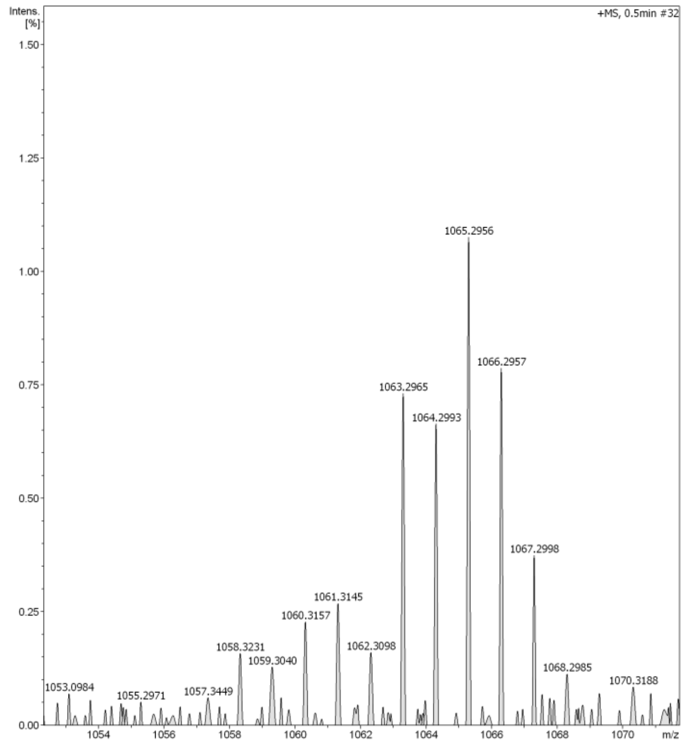

Figure S6 High-resolution mass spectrum of **IrL1** at room temperature.

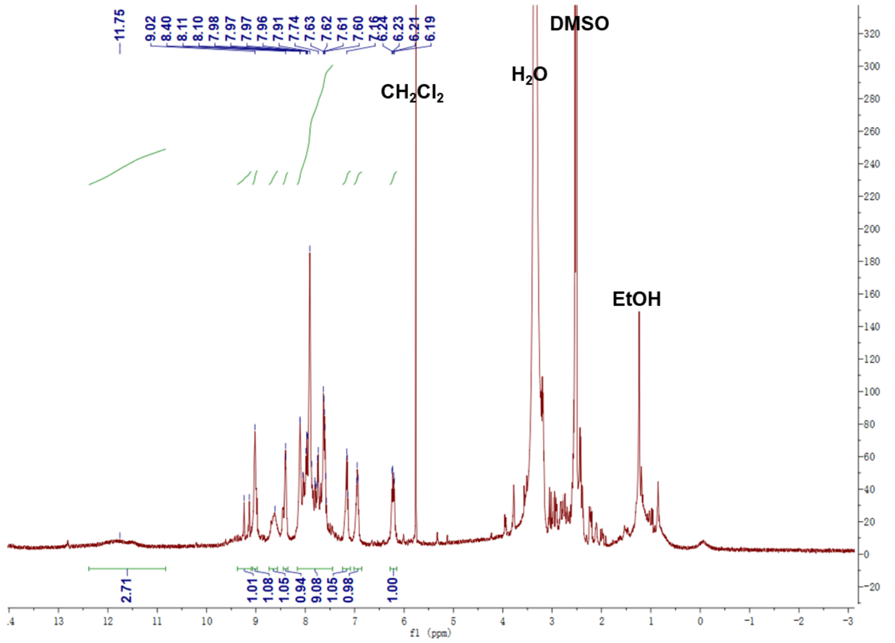

Figure S7 ^{1}H NMR spectrum of **IrL2** in DMSO-d_6 at room temperature.

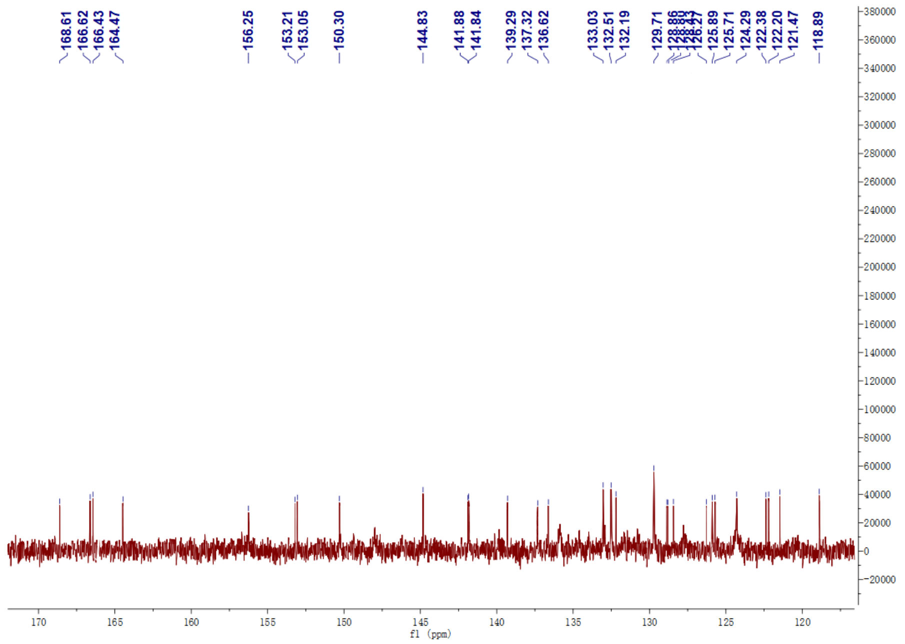

Figure S8 ^{13}C NMR spectrum of **IrL2** in DMSO-d_6 at room temperature.

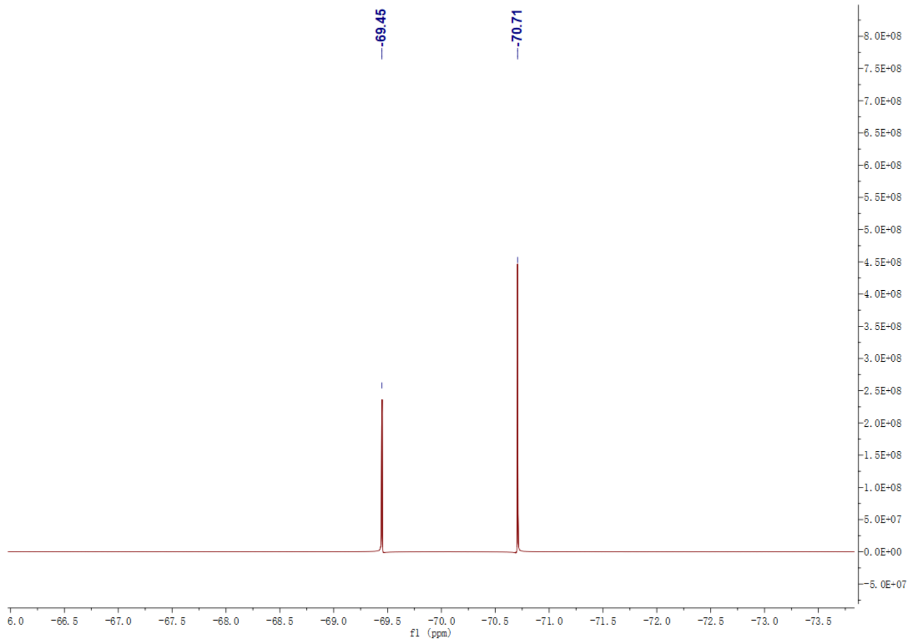

Figure S9 ^{19}F NMR spectrum of **IrL2** in DMSO-d_6 at room temperature.

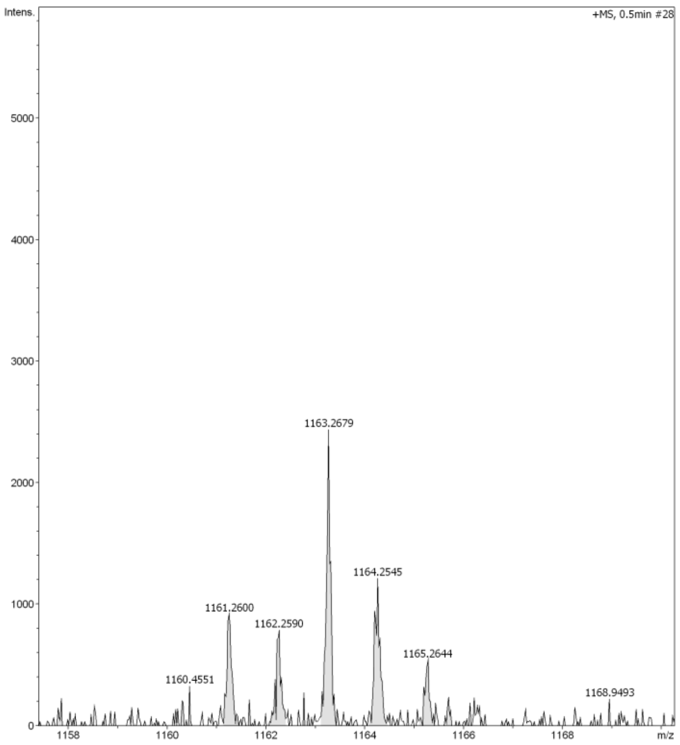

Figure S10 High-resolution mass spectrum of **IrL2** at room temperature.

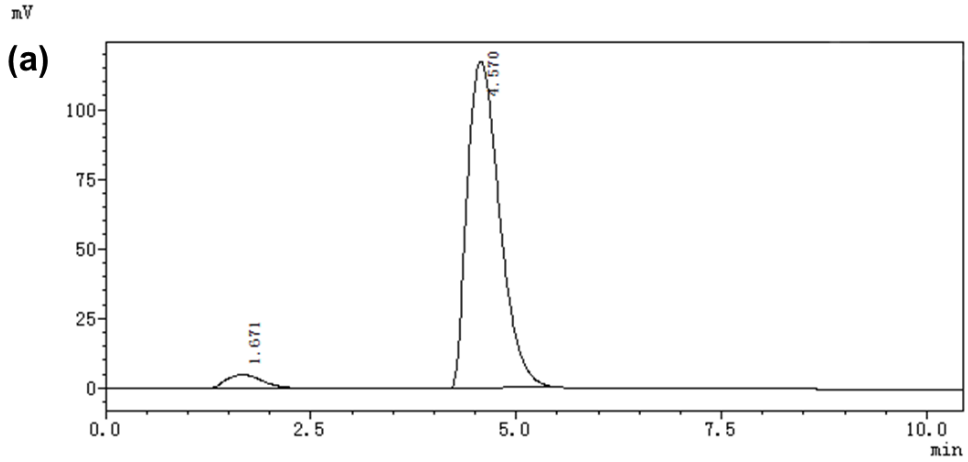

t_R/ min	Peak area	Compounds
**1.671**	**139692**	**MeOH**
**4.570**	**3211717**	**IrL1**

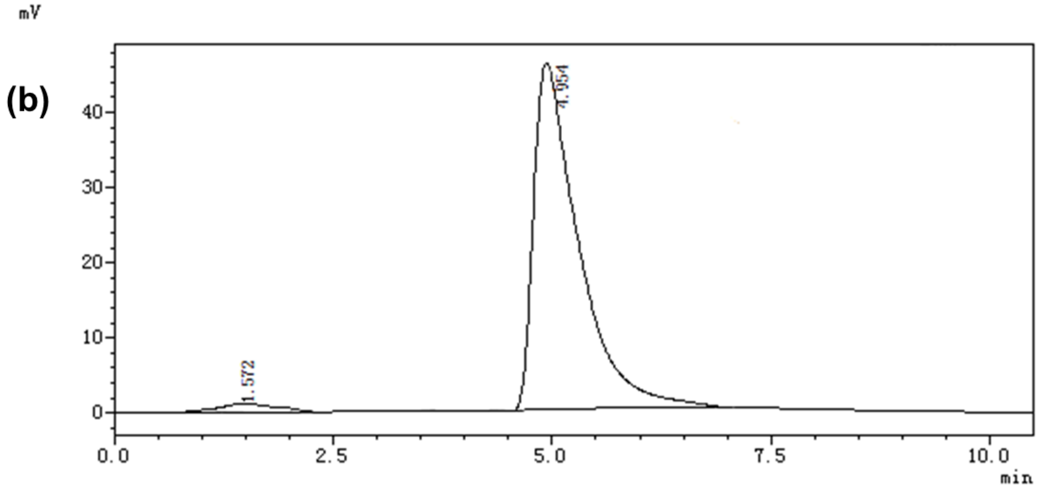

t_R/ min	Peak area	Compounds
**1.572**	**37055**	**MeOH**
**4.954**	**1679975**	**IrL2**

Figure S11 HPLC chromatograms of **IrL1** (a) and **IrL2** (b). Mobile phase: CH_3CN:H_2O = 3:2 (v:v). Chromatographic column: C18 column (4.6 mm × 150 mm, 5 μm).

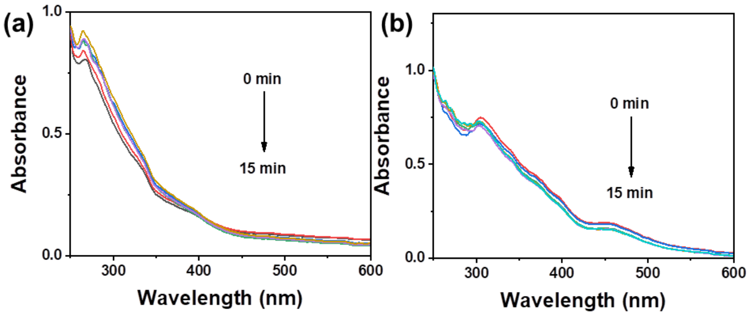

Figure S12 UV–vis absorption spectra of **Ir1** (a) and **Ir2** (b) upon exposure to LED light (425 nm, 20 mW cm^{-2}) in 15 min. Complexes concentration: 10 mM in mixed solvent (MeOH/H_2O =1/100, v/v).

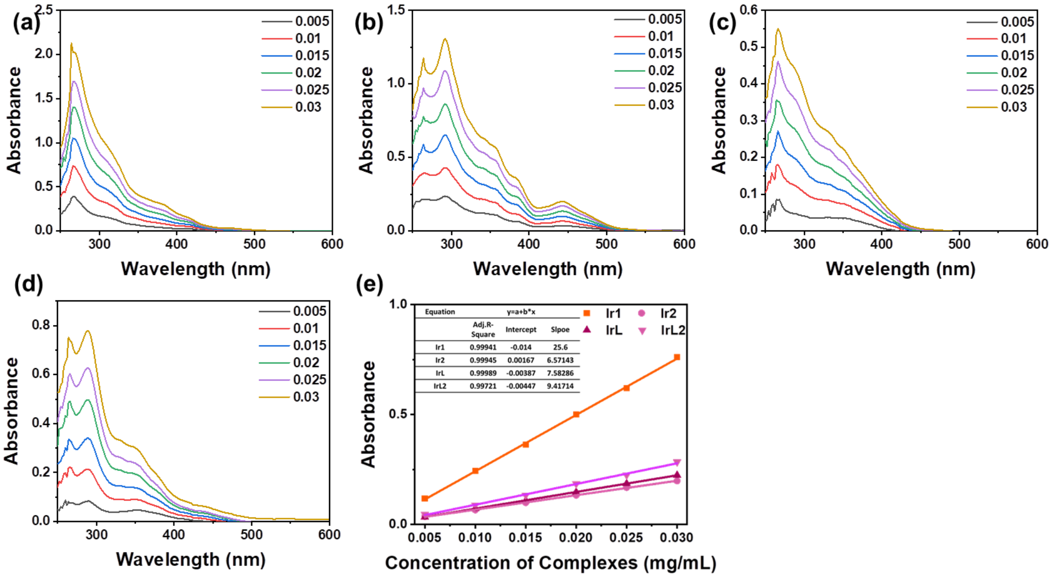

Figure S13 UV–vis absorption spectra of **Ir1** (a), **Ir2** (b), **IrL1** (c) and **IrL2** (d) in different concentrations (mg/mL) in DMF. (e) Standard curve of **Ir1**, **Ir2**, **IrL1** and **IrL2**.

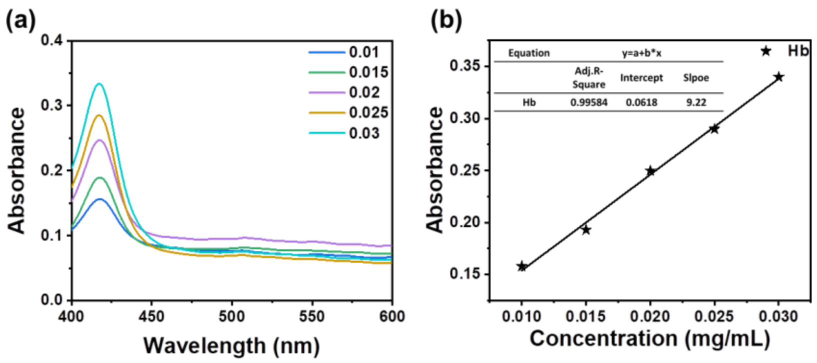

Figure S14 (a) UV–vis absorption spectra of Hb in different concentrations (mg/mL) in H_2O. (b) Standard curve of Hb.

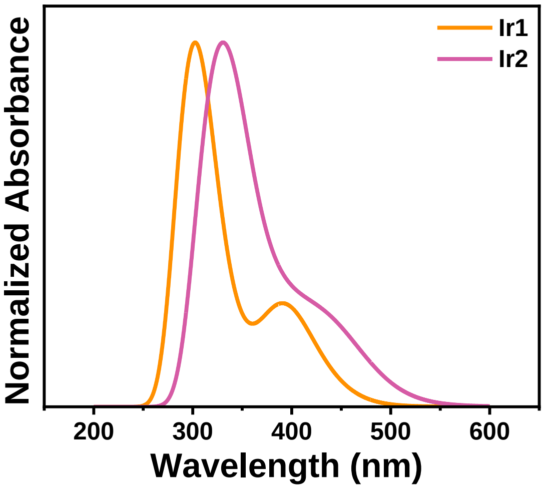

Figure S15 Theoretical absorption spectra of **Ir1** and **Ir2** in water based on DFT calculations.

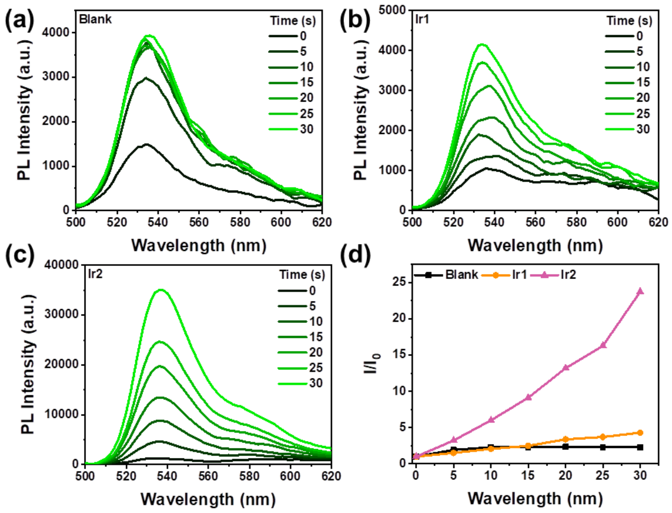

Figure S16 PL spectral changes of DCFH (a) upon exposure to LED light (425 nm, 20 mW cm^{-2}); in the presence of **Ir1** (b) and **Ir2** (c) upon exposure to LED light (425 nm, 20 mW cm^{-2}); (d) The change of PL intensity of DCFH with the change of time. I_0 = initial intensity of 525 nm. I = real-time intensity of 525 nm with various times of light exposure.

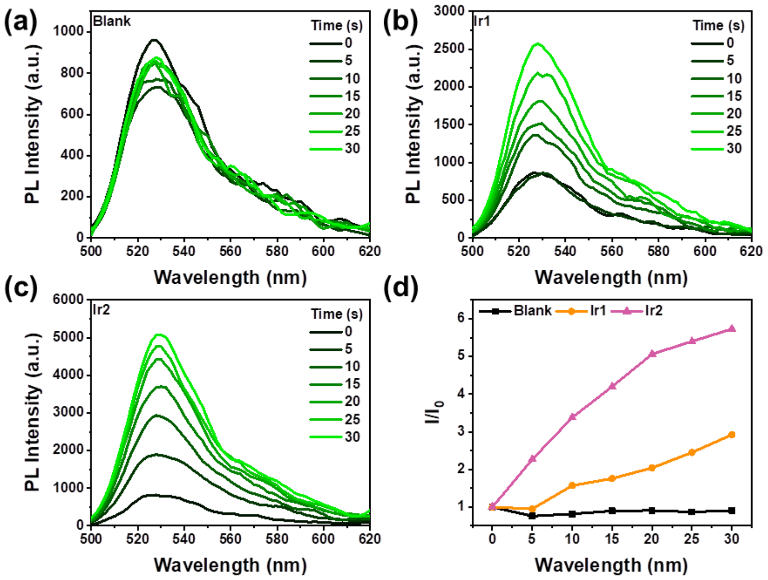

Figure S17 PL spectral changes of DHR 123 (a) upon exposure to LED light (425 nm, 20 mW cm^{-2}); in the presence of **Ir1** (b) and **Ir2** (c) upon exposure to LED light (425 nm, 20 mW cm^{-2}); (d) The change of PL intensity of DHR 123 with the change of time. I_0 = initial intensity of 525 nm. I = real-time intensity of 525 nm with various times of light exposure.

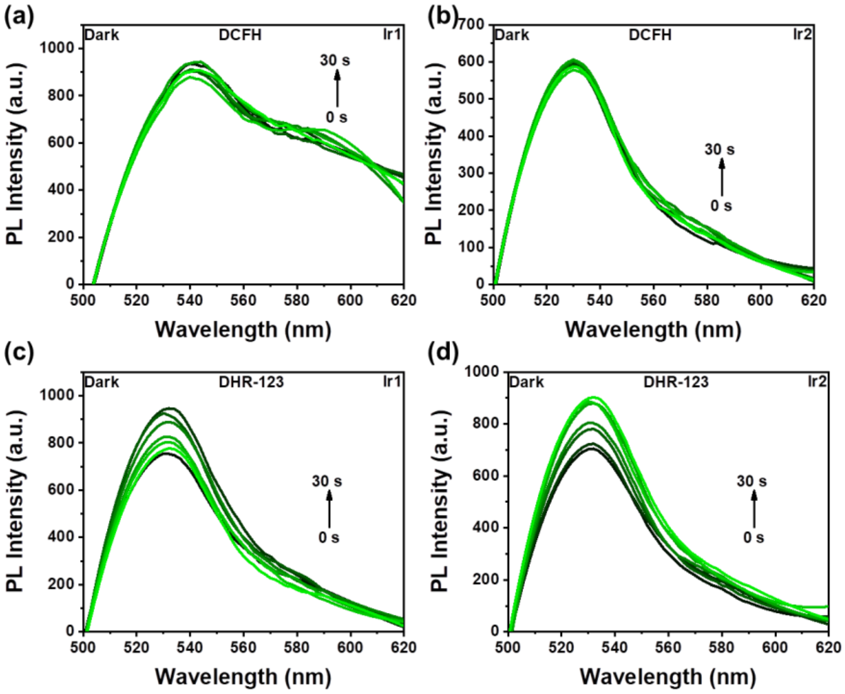

Figure S18 PL spectral changes of DCFH under darkness: in the presence of **Ir1** (a) and **Ir2** (b); PL spectral changes of DHR 123 under darkness: in the presence of **Ir1** (c) and **Ir2** (d). The spectra indicate that **Ir1** and **Ir2** have no effect on the indicators (DCFH and DHR 123).

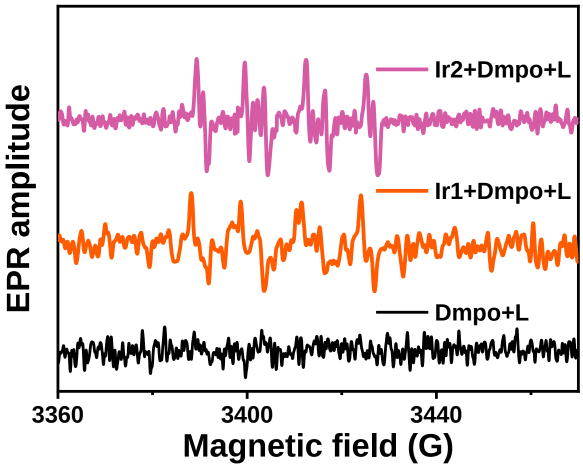

Figure S19 EPR signals of DMPO (for type-I ROS detection) with different treatments, as stated on the spectra.

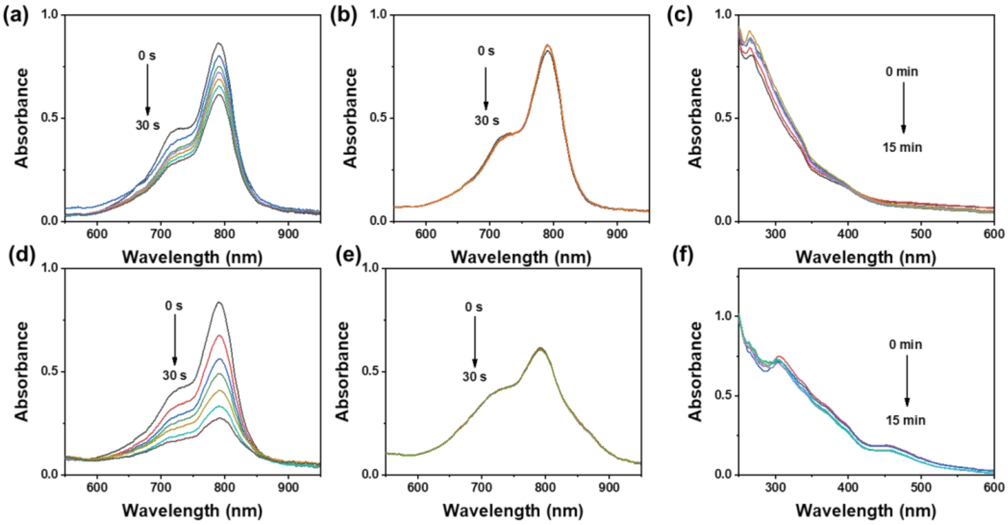

Figure S20 UV–vis absorption spectra changes of indocyanine green (ICG) (5 μg mL^{-1}) in the presence of **Ir1** (a), **Ir2** (d) upon exposure to LED light (425 nm, 20 mW cm^{-2}); in the presence of **Ir1** (b) and **Ir2** (e) under dark condition; UV–vis absorption spectra changes of **Ir1** (c) and **Ir2** (e) upon exposure to LED light (425 nm, 20 mW cm^{-2}) in 15 min.

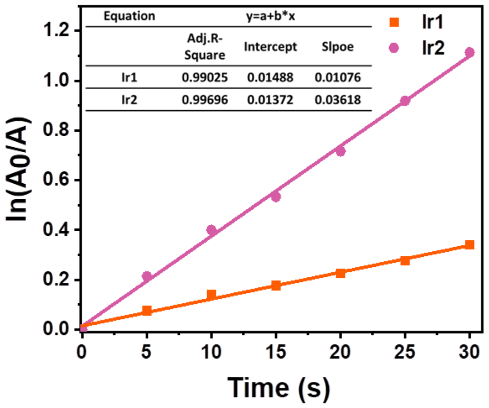

Figure S21 Time-dependent $^1O_2$ generation kinetics. A_0 = initial absorbance maximum of ICG. A = real-time absorbance maximum of ICG with various light exposures.

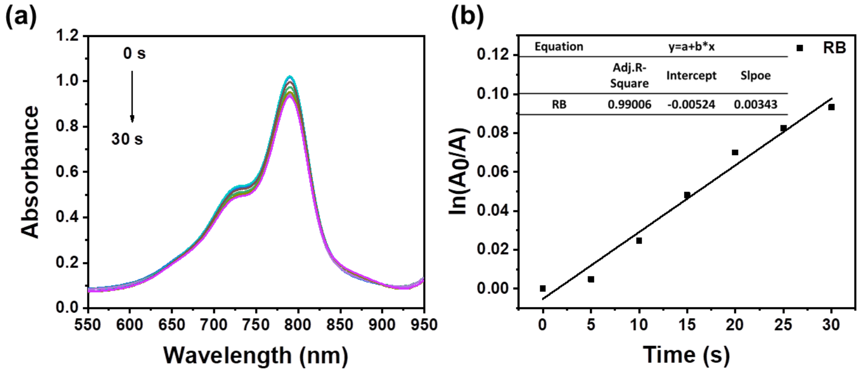

Figure S22 (a) UV–vis absorption spectra changes of indocyanine green (ICG) (5 µg mL^{-1}) in the presence of RB upon exposure to LED light (425 nm, 20 mW cm^{-2}). (b) Time-dependent $^1O_2$ generation kinetics of RB. A_0 = initial absorbance maximum of ICG. A = real-time absorbance maximum of ICG with various light exposures.

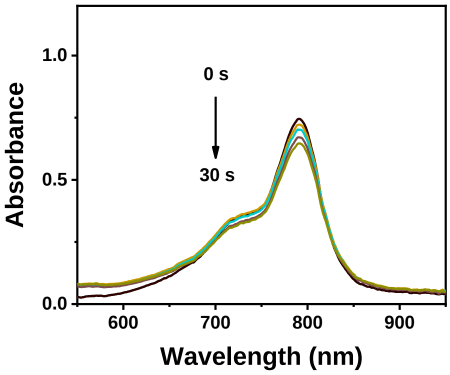

Figure S23 UV–vis absorption spectra changes of ICG (5 µg mL^{-1}) upon exposure to LED light (425 nm, 20 mW cm^{-2}).

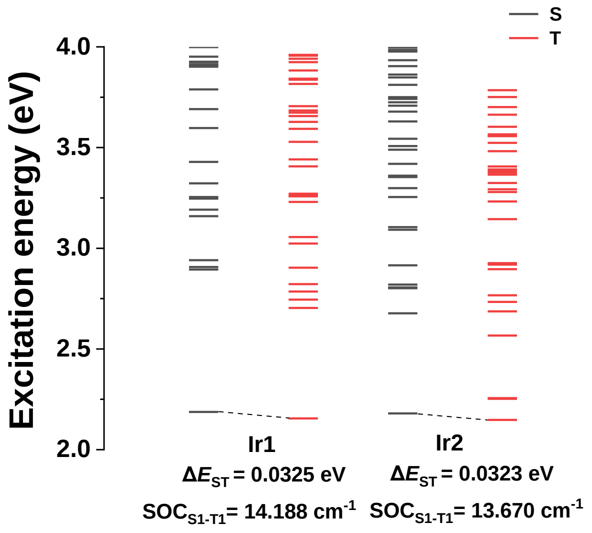

Figure S24 The single and triplet energy levels of **Ir1** and **Ir2** and their spin orbit coupling values with S_1-T_1 and S_0-T_1 transitions.

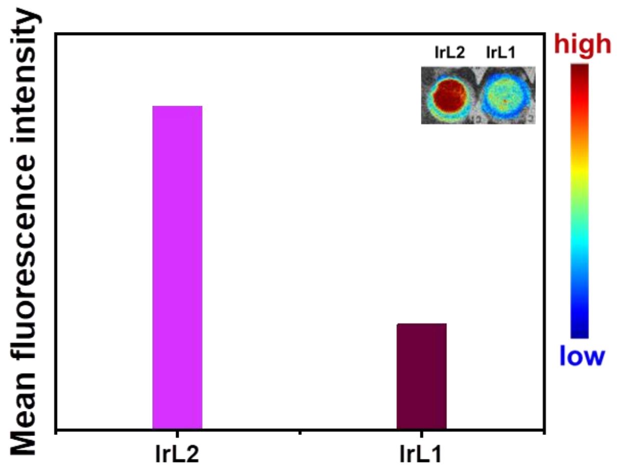

Figure S25 Average intensity of CL of **IrL2** and **IrL1** mixed with H_2O_2 (200 μM) and Hb (5 μg mL^{-1}) at pH = 7.4. Inset image: the CL images of **IrL2** and **IrL1**.

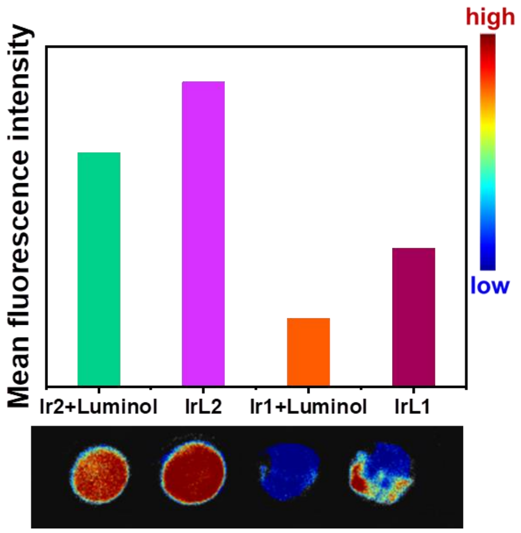

Figure S26 Average intensity of CL and CL images for the different samples. The covalently linked **IrL1** and **IrL2** exhibit stronger luminescence than simple (non-covalent) mixtures of **Ir1** and **Ir2** and luminol.

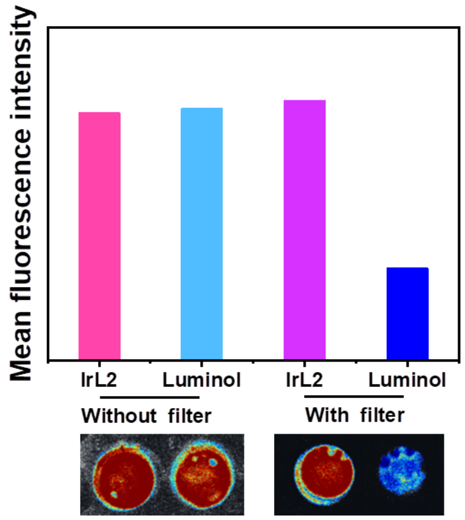

Figure S27 Average intensity of CL and CL images for the different samples with different treatments. The control experiment to detect the luminescence image of luminol molecules with or without the long pass 510 nm emission filter indicates that the CL of luminol cannot influence the detection of the **IrL2** CL images as the blue emission cannot pass the filter.

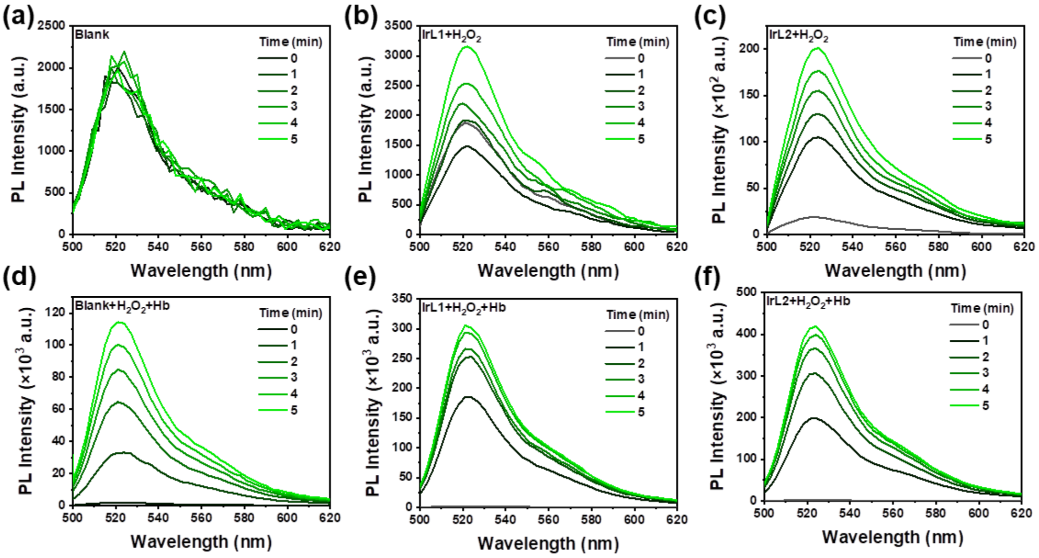

Figure S28 PL spectral changes of DCFH in different conditions. Solvent: DMF/H_2O=1/1000, v/v, pH= 6.5

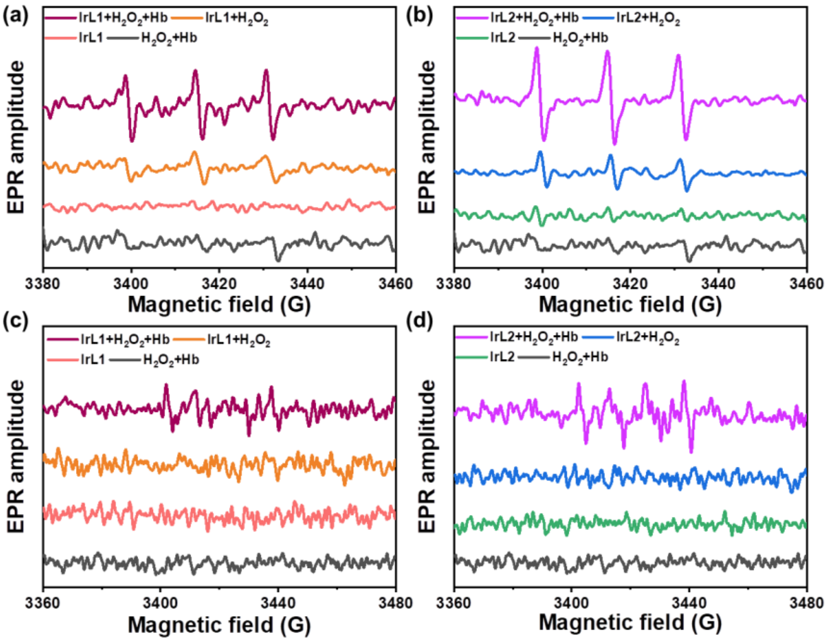

Figure S29 EPR signals of TEMP (for type II ROS detection) (a, b) and DMPO (for type I ROS detection) (c, d) under different conditions. Solvent: DMF/H_2O=1/1000, v/v, pH= 6.5.

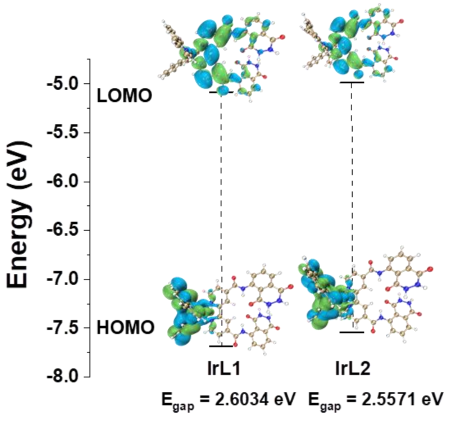

Figure S30 Energy level distribution and HOMO-LUMO energy gap in **IrL1** and **IrL2**.

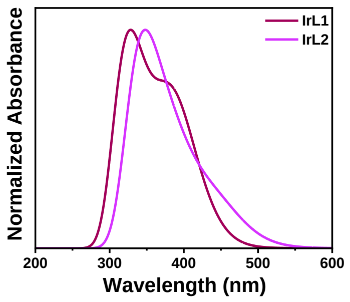

Figure S31 Theoretical absorption spectra of **IrL1** and **IrL2** in water.

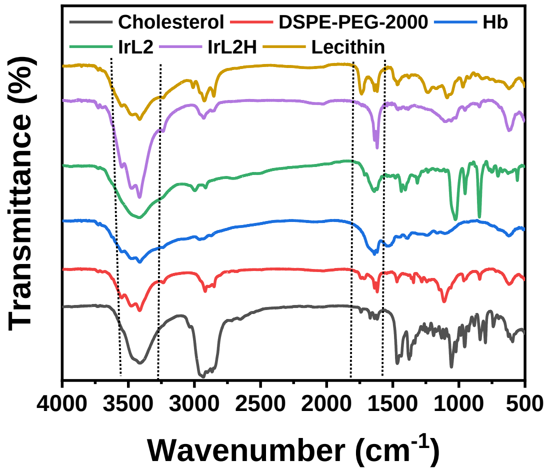

Figure S32 FT-IR spectra of cholesterol, lecithin, DSPE-PEG2000, **IrL2**, Hb, and **IrL2H**.

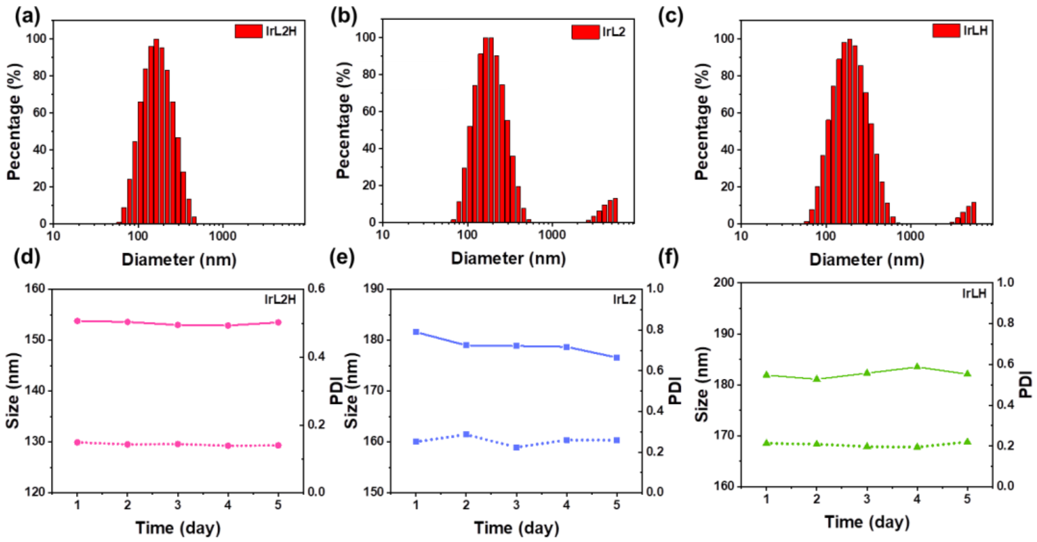

Figure S33 DLS data for the nanoparticles of **IrL2H** (a), **IrL2** (b) and **IrLH** (c) and the change of their size (solid lines) and PDI (dashed lines) (d-f).

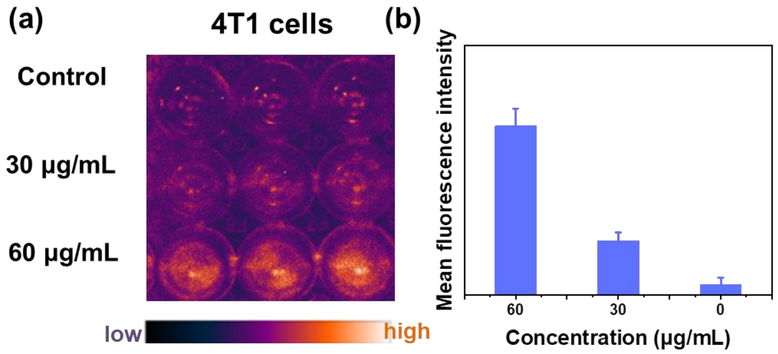

Figure S34 (a) CL image of **IrL2H** in 4T1 cells; (b) Average fluorescence intensity of CL with different treatments.

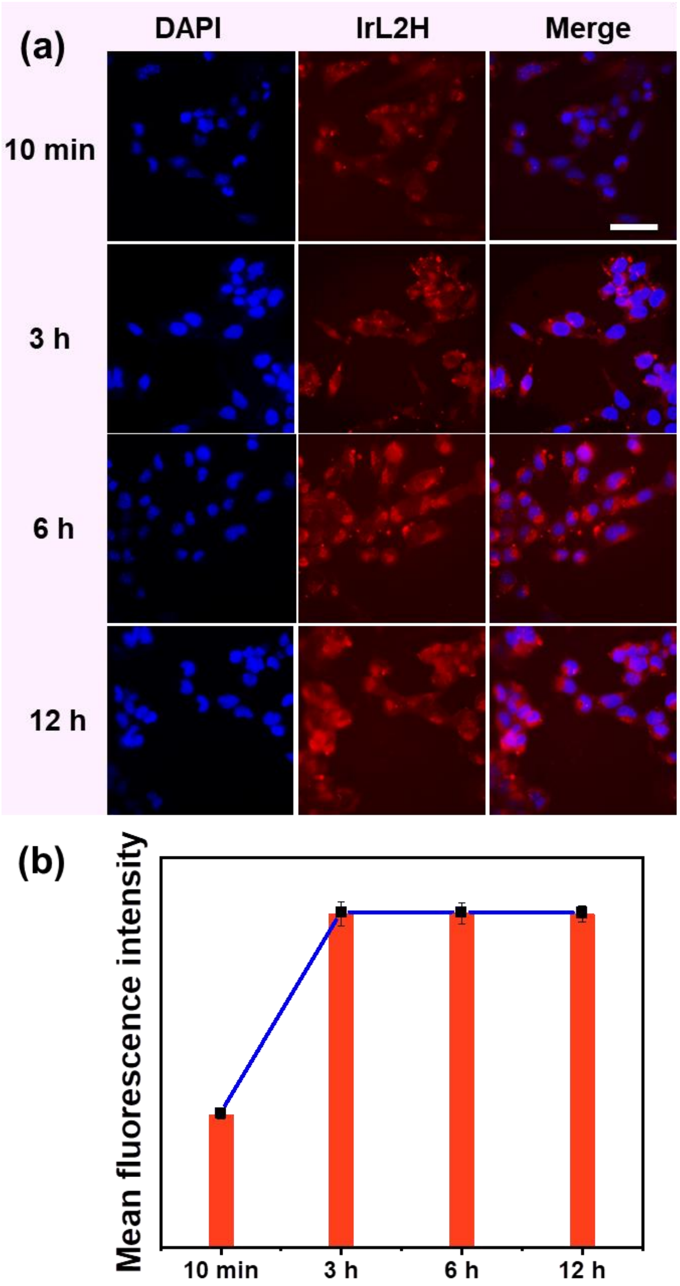

Figure S35 (a) CLSM images of 4T1 cells after incubation with **IrL2H** (concentration of Ir complex = 20 μM) for 10 min, 3 h, 6 h and 12 h. Scale bar = 50 μm. (DAPI = 4',6-diamidino-2-phenylindole). (b) The average fluorescence intensity of the red channel CLSM images of 4T1 cells after incubation with **IrL2H** for 10 min, 3 h, 6 h and 12 h.

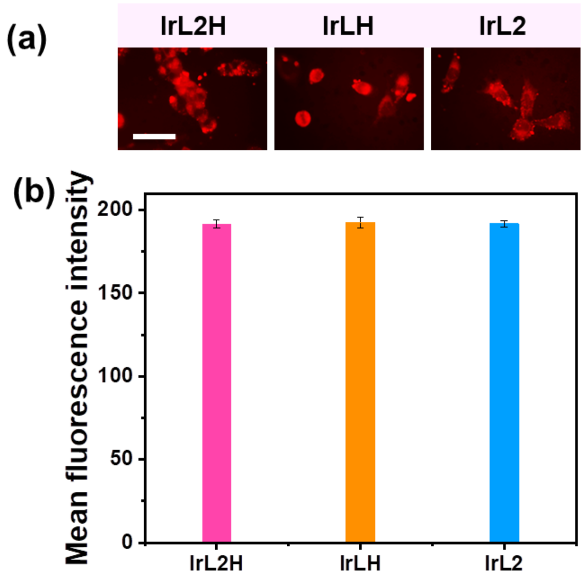

Figure S36 (a) CLSM images of 4T1 cells after incubation with **IrL2H**, **IrLH** and **IrL2** (concentration of Ir complex = 20 μM) for 3 h. Scale bar = 50 μm. (b) The average fluorescence intensity of the red channel CLSM images of 4T1 cells after incubation with **IrL2H**, **IrLH** and **IrL2** for 3 h. The results showed that there is no significant difference in the fluorescence from the Ir complexes at the same incubation time, which indicates little difference in the intracellular accumulation and suggests that **IrL2H**, **IrLH** and **IrL2** can be applied to the comparison of cytotoxicity.

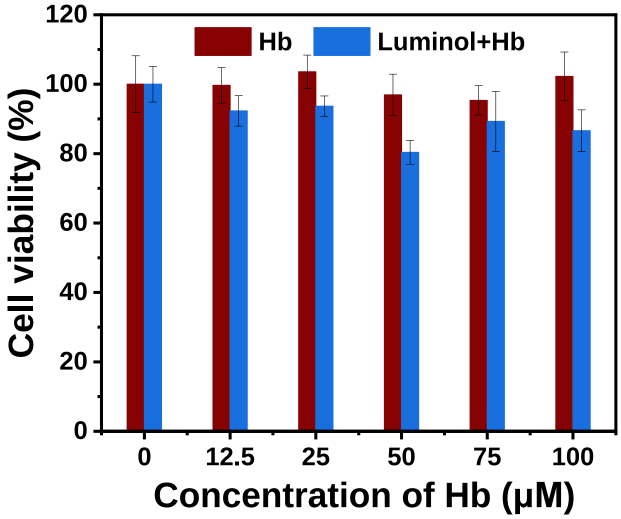

Figure S37 Relative viability of 4T1 cells after 24 h co-incubation with Hb and luminol+Hb.

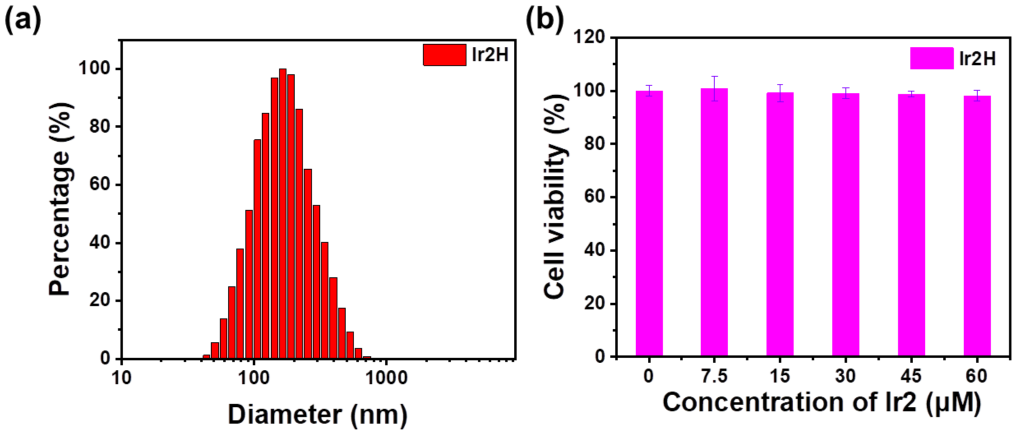

Figure S38 (a) DLS data for the nanoparticles of **Ir2H**. (b) Relative viability of 4T1 cells after 24 h co-incubation with **Ir2H**. The nanoparticles with liposomes containing **Ir2** and hemoglobin (named as **Ir2H**) also show almost no cytotoxicity to 4T1 cells.

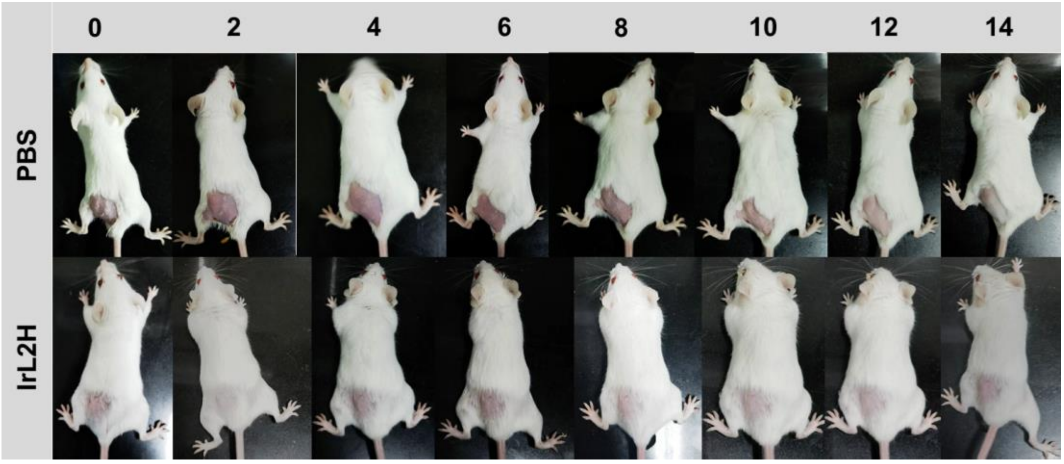

Figure S39 IrL2H-mediated inhibition of tumor growth in 4T1 tumor model. Photos of tumors after treatment.

Table S1. Photophysical data of **Ir1**, **IrL1, Ir2** and **IrL2**

	λ_{abs}[a] (nm)	λ_{em}[a] (nm)	Φ_p[a] (%)	τ_p[a] (ns)	k_r[b] ($\times10^6$ s^{-1})[b]	k_{nr}[b] ($\times10^7$ s^{-1})[b]
**Ir1**[a]	254, 325	615	12.69	90.66	1.40	9.63
**Ir2**[a]	291, 438	632	13.45	150.73	0.89	5.74
**IrL1**[c]	251, 303, 373	435, 620	14.99	48.33	3.10	17.59
**IrL2**[c]	288, 355, 439	440, 635	16.66	166.48	1.00	5.01

[a] Measured in MeOH (complex concentration = 1.0×10^{-5} M) at room temperature. [b] The radiative k_r and non-radiative k_{nr} values in neat film were calculated according to the equations: $k_r = \Phi / \tau$ and $k_{nr} = (1-\Phi)/ \tau$, from the quantum yields Φ and the lifetime τ values. [c] Measured in DMF (complex concentration = 1.0×10^{-5} M) at room temperature.
